# Nitrate reduction salvage pathway in *Methanococcales*

**DOI:** 10.3389/fmicb.2026.1824787

**Published:** 2026-07-06

**Authors:** Amelie Heidenreich, André G. Gouveia, Tristan Wagner

**Affiliations:** 1 Max Planck Institute for Marine Microbiology, Bremen, Germany; 2 Univ. Grenoble Alpes, CEA, CNRS, Institut de Biologie Structurale, Grenoble, France

**Keywords:** hyperthermophile, metabolic adaptation, methanogenic archaea, nitrate reduction, nitrite reductase

## Abstract

Nitrate is the most oxidized form of nitrogen and an essential nutrient for many living organisms. Its utilization was considered impossible in methanogenic archaea, since nitrate reduction inherently generates nitrite, a potent oxidant that can disrupt their catabolism. Yet, our study demonstrates that the hyperthermophile *Methanocaldococcus infernus* defies this rule by growing on nitrate as its sole nitrogen source. Comparative analyses revealed genes encoding a putative nitrate transporter and a nitrate reductase in *M. infernus*, as well as in *Methanothermococcus thermolithotrophicus*, which was first discovered to consume nitrate. The minimal operon is detected in many bacterial species inhabiting similar niches, supporting horizontal gene transfer acquisition. Based on *in silico* investigations, we propose that the transporter is a symporter that would rely on an ion gradient. We also predict that the putative nitrate reductase contains all molecular determinants for its activity. The observed nitrate-dependent growth in the absence of molybdenum would imply a tungsten-dependent nitrate reductase. The last reaction of the pathway is catalyzed by a F_420_H_2_-dependent sulfite reductase. The structure obtained at atomic resolution reveals an endogenous mixture of nitrite and sulfite bound to the siroheme catalyst, underscoring the enzyme’s dual function previously demonstrated *in vitro*. Our results led us to a metabolic model in which the nitrate-assimilation pathway would be indirectly powered by methanogenesis and H_2_-oxidation. This adaptation is another remarkable example of how *Methanococcales* extend their assimilation capabilities by hijacking bacterial systems and repurposing their F_420_H_2_-sulfite reductase to prevent oxidative damage.

## Introduction

Biological methane production by archaea accounts for half of the yearly atmospheric emissions of the greenhouse gas (Thauer et al., 2008; Liu and Whitman, 2008). This microbial process, fundamental to the degradation of biological matter, greatly contributes to Earth’s carbon cycling (Wagner et al., 2025). While methanogens are distributed worldwide, they are sequestered to anaerobic niches due to the extreme sensitivity of their catabolic machinery to oxidative damage despite detoxification systems (Jasso-Chávez et al., 2015; Tholen et al., 2007; Lyu and Lu, 2018). Among other oxidants, nitrate (NO_3_^−^) has been reported to negatively affect methanogenesis, which is of particular interest in the agricultural field for mitigating rumen methane production. NO_3_^−^ addition to the microbial community decreases methanogenic rates, either indirectly by stimulating hydrogen consumption by NO_3_^−^-reducing microbes, or directly by affecting methanogenic enzymes (Yang et al., 2016; Llonch et al., 2017). Previous work indicated that NO_3_^−^ growth inhibition is highly effective against the methylotrophic/acetoclastic *Methanosarcina barkeri* (e.g., inhibiting 50% of the methane production at 3 mM), the hydrogenotroph methanol-reducer *Methanosphaera stadtmanae* (i.e., complete inhibition at 33 mM), while the hydrogenotrophs *Methanospirillum hungateii* and *Methanobacterium bryantii* showed greater tolerance, with growth inhibition impacted at 15.3 mM for *M. hungateii* and a decrease of methane production by 50% at 25 mM for *M. bryantii* (Miller and Wolin, 1985; Belay et al., 1990; Klüber and Conrad, 1998). Other hydrogenotrophs belonging to *Methanobacterium* species, such as *Methanothermobacter thermautotrophicus*, and *Methanobrevibacter smithii*, showed even higher tolerance with no growth inhibition detected at 20 mM NO_3_^−^ (Belay et al., 1990). The most impressive case is *Methanothermococcus thermolithotrophicus* DSM 2095, a marine thermophile belonging to the *Methanococcales*, which shows exceptional tolerance to NO_3_^−^ (with growth still occurring at 203 mM NO_3_^−^ (Belay et al., 1990)). Even more striking, *M. thermolithotrophicus* has been shown to grow on NO_3_^−^ as the sole nitrogen source (Belay et al., 1990). NO_3_^−^ utilisation appears to be tungsten-dependent, as the absence of molybdenum did not dramatically inhibit the growth (Belay et al., 1990). However, the dependent NO_3_^−^ growth might require additional cellular energy demand as it reduces final yields (i.e., based on the absorbance at 600 nm, Abs_600nm_) by 20–25% compared to NH_4_Cl-dependent growth (Belay et al., 1990). The NO_3_^−^assimilation of *M. thermolithotrophicus* is unusual, and conflicts with two physiological aspects of methanogens: (i) an energy limitation due to the thermodynamic constraints of the hydrogenotrophic pathway, (ii) a catabolic metabolism sensitive to oxidation, with the activity of the methane-generating enzyme (methyl-coenzyme M reductase, MCR; Thauer, 2019; Duin et al., 2016; Lemaire and Wagner, 2022) being incompatible with the predicted intermediate nitrite (NO_2_^−^). Therefore, it is expected that the methanogen would rely on specific adaptive strategies if the complete CO_2_-reduction pathway is located in the same cellular compartment (e.g., the cytoplasm) as the NO_3_^−^-reduction pathway.

Metabolic pathways in which NO_3_^−^ undergoes reduction are divided into assimilation and dissimilation. The prokaryotic assimilatory route relies on cytoplasmic molybdopterin-dependent NO_3_^−^ reductase (NAS, belonging to the dimethylsulfoxide reductase family) to reduce NO_3_^−^ to NO_2_^−^, which is then further converted to ammonium (NH_4_^+^) by siroheme-containing NO_2_^−^ reductases (NirA, NirB) (Kuypers et al., 2018; Wang and Gunsalus, 2000). The electron donor of these reactions is either ferredoxin or NAD(P)H (Lin and Stewart, 1998; Fenn et al., 2021). In contrast, dissimilatory NO_3_^−^ reduction, mediated by membrane-bound NO_3_^−^ reductase (NAR) or periplasmic NO_3_^−^ reductase (NAP), produces NO_2_^−^ as part of the respiration (Kuypers et al., 2018; Stolz and Basu, 2002; Jepson et al., 2006). Downstream enzymes, such as cd₁- (including both *c*-type and *d_1_*-type heme) or Cu-containing NO_2_^−^ reductases (NirK, NirS) in denitrification, convert NO_2_^−^ to gaseous nitrogen compounds, while periplasmic cytochrome c nitrite reductase (ccNIR) reduces it to NH_4_^+^ (Almeida et al., 2003; Graf et al., 2014). However, the lack of cytochromes in hydrogenotrophic methanogens, such as *Methanococcales* (Costa and Whitman, 2023), would imply a cytoplasmic assimilatory NO_3_^−^ pathway to channel nitrogen into biomass.

In this work, we took advantage of recent studies on nitrogen assimilation in *Methanococcales* (Maslać et al., 2022) to elucidate the molecular basis of the NO_3_^−^ assimilation pathway. Through a combination of genome analyses, phylogeny, physiology, and biochemistry, we propose how NO_3_^−^ reduction can coexist with hydrogenotrophic methanogenesis and its origin by studying the enzymes that constitute the pathway.

## Methods

### Cultivation and physiology experiments

*Methanocaldococcus infernus* DSM 11812 (Leibniz Institute DSMZ - German Collection of Microorganisms and Cell Cultures, Braunschweig, Germany) was grown under anoxic conditions in minimal mineral medium prepared according to Maslać et al. (2022) with slight modifications: Na_2_SeO_3_ ·5H_2_O (2 μM final) instead of Na_2_SeO_4_; Na_2_WO_4_·2H_2_O of 100 μM final; trace element solution without Na_2_MoO_4_·2H_2_O; and pH adjusted to 6.5, similarly to Maslać et al. (2025a) and Maslać et al. (2025b). *M. infernus* was cultivated in 1 L pressure-resistant Duran bottles with 0.5 mM final concentration of Na_2_S as a reductant and sulfur source unless stated otherwise. The inoculum was added at a 1:10 ratio with the bottle headspace filled up to +1 bar H_2_/CO_2_ (mixture of 80:20) and incubated at 75 °C in the dark, standing without shaking. Anoxic nitrogen-free medium (absence of N_2_ or NH_4_Cl from base medium) was used for physiological experiments, in which NaNO_3_ or NH_4_Cl was added as a sole nitrogen source. Nitrogen-source competitive assimilation experiments were performed by simultaneously adding 5 mM NaNO_3_ and 5 mM NH_4_Cl. NO_3_^−^ as the sole source was screened at concentrations ranging from 0 to 100 mM in a 200 mL serum bottle containing 10 mL of medium (with 2 μM Na_2_SeO_4_ and 1 mM Na_2_S). Growth measurements were performed using the UVmini-1240 (Shimadzu, Germany) at 600 nm, and culture gas phases were refreshed at least once.

A 2 L glass vessel fermenter filled with 1.4 L of medium was used for large-scale biomass production. The medium is the same as previously described, with a final concentration of 5 μM Na_2_SeO_3_ ·5H_2_O and 22 mM NaNO_3_. The pH was kept at 6.5 using a KOH or HCl concentrated solution. The culture was maintained in a reduced state by the continuous addition of a solution of 540 mM Na_2_S (also serving as a sulfur source) at a flow rate of 0.7 mL/h for the initial 4–6 h, and increased to 1.4 mL/h. This resulted in a progressive increase in pH, which was counterbalanced by HCl addition. After 7–9 h of cultivation, Na_2_S input was increased to 2.1 mL/h. When the culture reached an Abs_600nm_ between 1.5 and 2.3, cells were anaerobically harvested at 6,000 x *g* for 30 min at 20 °C in an atmosphere containing N_2_/CO_2_ (at a 90:10 ratio) and subsequently transferred to a pressure-resistant bottle with +0.5 bar of N_2_ and stored at −80 °C. On average, one preparation yields 3–4 g of wet-weight cells.

### NH_4_^+^ concentration measurements

NH_4_^+^ concentrations were measured using the salicylate–nitroprusside colorimetric method (Maslać et al., 2022). Samples were obtained by aliquoting 0.5 mL of growth culture at each time point aerobically, followed by a centrifugation for 5 min at 15,700 x *g*. The following procedure was performed aerobically. In brief, 40 μL of sample or NH_4_Cl standard was mixed with 80 μM of salicylate reagent (424.7 mM sodium salicylate, 193.8 mM tri-sodium citrate dihydrate, 193.8 mM di-sodium tartrate dihydrate and 0.95 mM sodium nitroprusside dihydrate, freshly prepared) with 80 μM of hypochlorite reagent (10% sodium hypochlorite and 1.5 M NaOH mixed in the ratio of 1:36). Samples were incubated at room temperature for 45 min in the dark to allow color development. The indophenol complex formed was quantified by measuring absorbance at 650 nm using an Infinite 200 PRO plate reader (Tecan, Switzerland). A standard curve was generated from serial dilutions of NH_4_Cl (0–600 μM), and concentrations were calculated by linear regression. All standards were measured in technical triplicate, and blanks containing distilled water were included in each run to correct for background absorbance.

### NO_3_^−^/NO_2_^−^ measurements

NO_3_^−^ and NO_2_^−^ concentrations were measured using the MQuant™ colourimetric test strips (Merck, Germany). Standards (0–10 mM) were prepared by diluting 1 M NO_3_^−^ and NO_2_^−^ stock solutions (sodium salts, Sigma Aldrich, Germany) into the cultivation medium. Sample concentrations fell within the standard range and were analyzed without dilution, thereby minimizing potential handling error. For each assay, 5 μL of standard or sample was applied to the test strip and incubated for 8 min at room temperature. Strips were scanned (PDF and TIFF formats), and a maximum of nine were processed per batch. Color intensity was quantified by extracting red, green, and blue (RGB) values from a 30-pixel region around a manually selected central point on each pad using an in-house R script. Only red channel values correlated with concentration were used to generate a standard curve for interpolating sample values.

### Protein purification

NO_3_^−^-grown cells obtained from a fermenter were thawed at room temperature (with 8 g of wet weight cells used per preparation) and transferred into a N₂/CO₂ (at a 90:10 ratio) anaerobic tent. Lysis was performed via osmotic shock by adding 4 volumes of buffer A (50 mM Tris–HCl, pH 8.0, 2 mM dithiothreitol (DTT)) to 1 volume of thawed cells. The lysate was homogenised and further processed by sonication (Sonopuls, Bandelin Electronic, Germany) for 5 cycles (30 s on, 1 min off) at 70% amplitude, followed by centrifugation at 45,000 × g for 40 min at 20 °C (JA-25.5 rotor, Beckman Coulter). The supernatant was transferred into a N₂/H₂ (at a 97:3 ratio) Coy anaerobic chamber, filtered through a 0.2 μm membrane (Sartorius, Germany), and loaded onto a 5 mL HiTrap Q HP column (Cytiva, Germany) equilibrated with buffer A. Unbound proteins were washed with 10 mL buffer A, and the F_420_H_2_-dependent sulfite/NO_2_^−^ reductase from *M. infernus* (*Mi*Fsr) eluted between 100 and 700 mM NaCl gradient over 70 min at 2 mL/min. Fractions were collected and analyzed via absorbance at 280, 415, and 590 nm. Fractions of interest were pooled based on activity assay and denaturing electrophoresis (SDS–PAGE) for each chromatography step. The merged pool was diluted 1:2 with buffer A, and reloaded in a second 5 mL HiTrap Q HP column equilibrated with buffer A. Elution was performed with a 0–50% gradient of buffer C (500 mM malate pH 5.0, 2 mM DTT) over 60 min at 1.5 mL/min. Pooled fractions containing *Mi*Fsr were diluted with a 1:10 ratio of HIC buffer (50 mM Tris–HCl pH 8.0, 1.5 M (NH_4_)_2_SO_4_, and 2 mM DTT) and loaded onto a HiTrap Phenyl-Sepharose HP 5 mL column (Cytiva, Germany) equilibrated with HIC buffer. Unbound proteins were washed with 10 mL HIC buffer, and the elution was carried out with a 1.5–0 M (NH₄)₂SO₄ gradient over 60 min at 1.2 mL/min. *Mi*Fsr was concentrated with a 3 kDa cut-off centrifugal filter to 300 μL prior to loading onto a Superose 6 10/300 GL column. The size-exclusion chromatography column was equilibrated in the storage buffer (25 mM Tris HCl pH 8, 2 mM DTT, and 10% glycerol (v/v)) and eluted at 0.4 mL/min.

### Activity measurements

NO_3_^−^ and NO_2_^−^ reductase activities were assayed by monitoring the oxidation of reduced benzyl viologen (BV). The 200 μL reaction mix contained 2 mM BV, 0.2 mM sodium dithionite, 5 mM substrate (NaNO_3_ or NaNO_2_), and buffer A. Selected fractions obtained during purification were diluted 1:5 or 1:10 in the reagent, depending on the protein concentration, as estimated from absorbance at 280 nm. Absorbance at 500 and 650 nm was recorded at 50 °C for screening fractions during purification and 65 °C for characterization using a FLUOstar Omega microplate reader (BMG Labtech, Germany). Activities were calculated from the maximal linear decrease in absorbance after subtraction of control slopes, applying the Beer–Lambert law (εBV = 7,600 M^−1^ cm^−1^). Experiments were performed in triplicate for the O_2_ sensitivity and in duplicate for fraction screening and F_420_H_2_-oxidase activity.

For the O_2_ sensitivity assay: *Mi*Fsr diluted to 65 μg/mL in buffer A was removed from the anaerobic chamber (containing 100% N_2_) and exposed to O_2_ for 10, 20, and 85 min, before being transferred back to the anaerobic chamber. The assay was run in a final volume of 200 μL with an anaerobically prepared reaction mix containing the following final concentration: 2 mM BV, 0.2 mM sodium dithionite, 2 mM NaNO_2_, 1.6 μg/mL of *Mi*Fsr, 38.5 mM Tris/HCl pH 8.0 and 1.5 mM DTT. The reaction was measured at 65 °C for 35 min, with one point recorded every 1 min 28 s.

For the F_420_H_2_-dependent activity: The F_420_ purification from *Methanothermococcus thermolithotrophicus* and its reduction by sodium borohydride were described in references (Jespersen et al., 2023; Deppenmeier et al., 1990). The assay was run in a final volume of 205 μL with a reaction mix containing a final concentration of 140 μM F_420_H_2_, 1 mM NO_2_^−^ or sulfite (SO_3_^2−^), 4.7 μg/mL of *Mi*Fsr (diluted in buffer A), and 85 mM KPO_4_ buffer at pH 7.0. The reaction was recorded at 419 nm and incubated at 65 °C.

### Crystallization

Prior to crystallization, the fresh sample was centrifuged at 13,000 x *g* for 3 min to remove macro-aggregates and dust. It must be noted that during the final concentration, the protein tends to aggregate on the filter as a black precipitate. The aggregate was partially resuspended, and the final protein concentration of 6.5 mg/mL might be underestimated. The sample was crystallized in an anaerobic chamber filled with N_2_/H_2_ (at a 97/3 ratio) at 20 °C. The crystallization was done in 96-Well MRC 2-Drop polystyrene plates (SWISSCI) containing 90 uL of crystallization solution in the reservoir. 0.7 uL of enzyme at a concentration of 6.5 mg/mL containing 1 mM FAD was mixed with 0.7 uL of the crystallization solution. The crystallization solution is made of 45% w/v pentaerythritol propoxylate (5/4 PO/OH), 100 mM 2-(*N*-morpholino)ethanesulfonic acid (MES) pH 6.5, and 400 mM potassium chloride for *Mi*Fsr^As^, and 45% w/v pentaerythritol propoxylate (5/4 PO/OH), 100 mM sodium acetate pH 4.6, and 400 mM potassium chloride for *Mi*Fsr^NO2^ (see Table 1). The dark brown crystals appeared in 7 to 12 days. Prior to flash-freezing in liquid nitrogen, *Mi*Fsr^NO2^ crystals were soaked in the crystallization solution supplemented with 50 mM NaNO_2_ for 5 min.

**Table 1:** X-ray data collection and refinement statistics.

	*M. infernus* Fsr as-isolated (*Mi*Fsr^As^)	*M. infernus* Fsr soaked with NO_2_^−^ (*Mi*Fsr^NO2^)
Data collection		
Wavelength (Å)	1.03320	1.03320
Space group	I222	I222
Resolution (Å)	87.60–1.58 (1.71–1.58)	86.86–1.21 (1.33–1.21)
Cell dimensions		
a, b, c (Å) / α, *β*, *γ* (°)	102.20, 114.11, 136.71 90, 90, 90	101.76, 113.00, 135.80 90 90 90
R_merge_(%)^a^	12.4 (195.7)	6.7 (93.8)
R_pim_ (%)^a^	3.5 (54.4)	3.1 (51.3)
CC_1/2_^a^	0.998 (0.558)	0.998 (0.470)
I/σ*_I_*^a^	11.4 (1.5)	11.9 (1.6)
Spherical completeness (%)^a^	81.8 (19.4)	73.2 (14.7)
Ellipsoidal completeness (%)^a^	94.8 (52.6)	95.1 (56.9)
Redundancy^a^	13.5 (13.8)	5.4 (4.2)
Nr. unique reflections^a^	89,629 (4,481)	175,298 (8,765)
Refinement		
Resolution (Å)	42.32–1.58	42.02–1.21
Number of reflections	89,621	175,289
R_work_/R_free_^b^ (%)	13.80/16.72	11.57/14.27
Number of atoms	5,881	11,252
Protein	5,031	10,241
Ligands/ions	233	222
Solvent	617	789
Mean B-value (Å^2^)	33.15	20.51
Molprobity clashscore, all atoms	0.47	0.38
Ramachandran plot		
Favored regions (%)	97.71	97.87
Outlier regions (%)	0	0.16
rmsd^c^ bond lengths (Å)	0.011	0.013
rmsd^c^ angles (°)	1.314	1.481
PDB ID code	9TN7	9TN9

^a^Values relative to the highest resolution shell are within parentheses. ^b^ R_free_ was calculated as the R_work_ for 5% of the reflections that were not included in the refinement. ^c^ rmsd, root mean square deviation. For Fsr soaked with nitrite the reported number of atoms, and the deposited structure contains hydrogens except on waters and ligands.

### X-ray data collection and structural analysis

The diffraction experiments used for the deposited models were performed at 100 K on the beamline PROXIMA-1 from the SOLEIL (Source Optimisée de Lumière d’Énergie Intermédiaire du LURE) synchrotron. The data were processed and scaled with *autoPROC* (Vonrhein et al., 2011; Vonrhein et al., 2024). The data presented a slight anisotropy and were further processed with *STARANISO* correction integrated with the *autoPROC* pipeline (Vonrhein et al., 2011; Vonrhein et al., 2024) (*STARANISO*; Tickle et al., 2016). Based on the sample biochemical properties, the crystal identity was presumed to be the F_420_H_2_-dependent sulfite reductase, and the structure was solved by molecular replacement using the PDB 7NP8 (Jespersen et al., 2023) as a template for Phaser from the PHENIX package (Liebschner et al., 2019). The model was manually built *via COOT* (Emsley et al., 2010) and refined with *phenix.refine* (Liebschner et al., 2019). *Mi*Fsr^As^ was refined by applying translation, libration, and screw-rotation. Instead, *Mi*Fsr^NO2^ refinement was performed by considering all atoms anisotropic. Both models were refined by adding hydrogens to the riding positions and validated using the MolProbity server (Chen et al., 2010). Data collection and refinement statistics for the deposited models are listed in Table 1. All figures with structures were generated and rendered with PyMOL (Schrödinger, LLC, New York, NY, United States).

### Phylogenetic analyses

The putative NO_3_^−^ reductase sequence from *Methanocaldococcus infernus* (WP_013099975.1) was used as a query in BLASTp searches against the RefSeq protein database. The top 500 homologous sequences were retrieved based on sequence similarity and alignment coverage. To assess functional specificity and discriminate NO_3_^−^ reductases from closely related molybdoenzymes, three representative formate dehydrogenase sequences were independently used as BLASTp queries against the RefSeq database, and the top 100 homologous sequences were retrieved. Sequence redundancy was reduced using the CD-HIT (Li and Godzik, 2006) web server on galaxy pasteur.fr (https://galaxy.pasteur.fr), applying an 80% sequence identity threshold, with one representative retained per cluster. Experimentally validated NO_3_^−^ reductase sequences from *Desulfovibrio* sp. (PDB ID: 2JIO) (Najmudin et al., 2008) and *Synechococcus elongatus* (Hirasawa et al., 2004) were included as references. TorA from *Escherichia coli* was used as an outgroup. *Methanothermococcus thermolithotrophicus* DSM 2095 corresponding genomic nucleotide sequence was retrieved and translated *in silico* into its amino acid sequence. The translated sequence, including the stop codon, was retained and used in the phylogenetic analysis. Protein sequences were aligned using MUSCLE with default parameters (Madeira et al., 2024). Maximum-likelihood phylogenetic reconstruction was performed using IQ-TREE 3 (Wong et al., 2026), with the best-fitting substitution model selected automatically using ModelFinder. Branch support was evaluated using 1,000 ultrafast bootstrap replicates (Kalyaanamoorthy et al., 2017; Hoang et al., 2018). Phylogenetic trees were visualized and annotated using iTOL (Letunic and Bork, 2024).

For phylogenetic analysis of the NO_3_^−^ transporter, the *M. infernus* sequence (WP_013099974.1) was used as a query in BLASTp searches against the RefSeq database, and the top 40 homologous sequences were retrieved. Additional NarK-type transporter sequences were manually included (Supplementary File S1: Figure 4). SulP from *Pseudomonas* was used as an outgroup. Maximum-likelihood phylogenetic inference was performed as described above, with branch support assessed using 1,000 bootstrap replicates.

## Results

### By losing its ability to assimilate NO_3_^−^, *M. thermolithotrophicus* hints at the enzymes composing the pathway

Previous comparative transcriptomic studies of *M. thermolithotrophicus* DSM 2095 under NH_4_Cl-fed culture conditions versus N_2_-fixation highlighted the expected upregulation of the genes involved in nitrogen fixation and deprivation (e.g., nitrogenase) (Maslać et al., 2022). Interestingly, it also revealed a 5 times log2 fold change upregulation of an operon encoding a putative NO_3_^−^ transporter, NO_3_^−^ reductase, and 2 times log2 fold change for a F_420_H_2_-dependent sulfite reductase (Fsr). Given its upregulation and the putative function of the encoded genes, this operon emerged as a candidate for the NO_3_^−^ utilizing pathway. Therefore, we hypothesized that the three genes would be the key actors of the pathway to (i) transport NO_3_^−^ to the cytoplasm, (ii) reduce NO_3_^−^ to NO_2_^−^, and (iii) further reduce NO_2_^−^ to NH_4_^+^.

**Figure 1:**
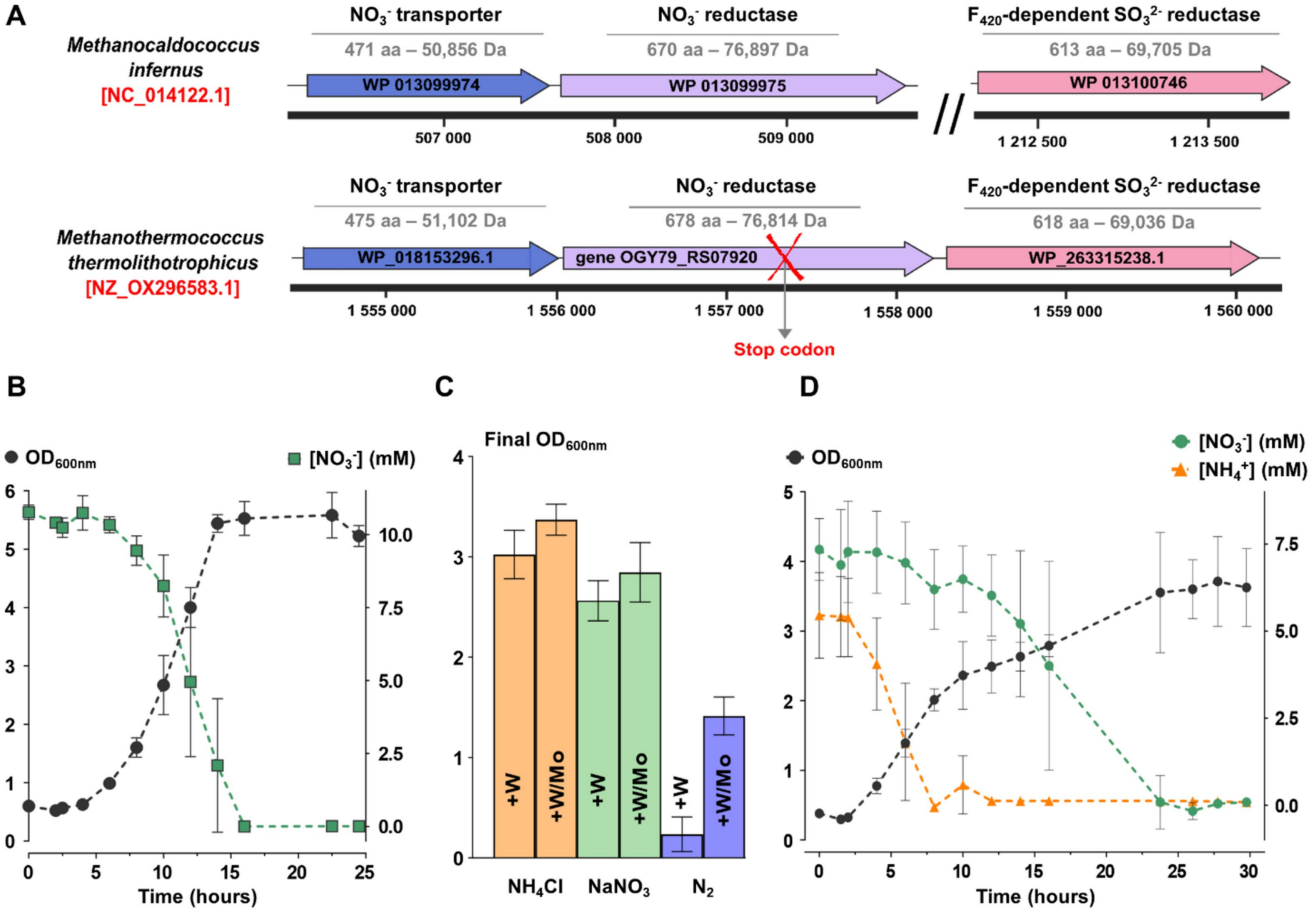
Growth of *M. infernus* under NO_3_^−^ source and influence of trace metal availability. **(A)** Arrangement of the putative NO_3_^−^ utilisation genes in *M. infernus* and *M. thermolithotrophicus.* **(B)** Growth curve of *M. infernus* cultures on 10 mM NO_3_^−^ (black circles) and NO_3_^−^ consumption during growth (green squares). **(C)** Molybdenum-free and molybdenum-supplemented *M. infernus* growth after 30 h under 24 mM NH_4_Cl (orange), 10 mM NaNO₃ (green), or N_2_ (blue) as nitrogen sources. **(D)** Growth curve of *M. infernus* cultures (black circles) co-supplied with NaNO₃ and NH₄Cl. NO_3_^−^ vs. NH₄Cl consumption was monitored during growth (green circles and orange triangles, respectively).

Despite our intensive cultivation efforts with our homemade optimized medium (Maslać et al., 2025b) or the original medium from Belay et al. (1990), we did not detect any growth when NO_3_^−^ was used as the sole nitrogen source. A more detailed inspection of the operon provided a potential explanation for this phenotype, as the putative NO_3_^−^ reductase gene revealed a premature stop codon. This likely disrupts gene function that would render NO_3_^−^ assimilation non-functional (Figure 1A). We then specifically searched the RefSeq database for closely related homologues that encode a full-length NO_3_^−^ reductase gene (i.e., lacking the disruptive stop codon) to argue that the integrity of this gene is a critical requirement for effective NO_3_^−^ utilization by methanogens. Of the homologues identified, only two species harbored operons similar to *M. thermolithotrophicus* DSM 2095 and did not display any putative disruptive mutation, representing potential candidates for complete NO_3_^−^ assimilation (Supplementary Figures S1A,B): *Methanocaldococcus indicus* and *Methanocaldococcus* sp. FS406-22. A third one, *Methanocaldococcus infernus* DSM 11812, harbors the operon without the *fsr* gene (Figure 1A). All of them are hyperthermophiles with the genetic potential for diazotrophy. For experimental validation, *M. infernus* was selected since our laboratory already uses this hydrogenotroph to characterize its N_2_-fixation pathway (Maslać et al., 2025a; Maslać et al., 2025b).

### *M. infernus* NO_3_^−^-dependent growth has similar properties to those previously described in *M. thermolithotrophicus*

As suspected, *M. infernus* was capable of growing on NaNO_3_ as the sole source of nitrogen (Figures 1B,C). The final biomass yield (Abs_600nm_) under NO_3_^−^ is reduced by ≈16% when compared to NH_4_Cl-dependent growth. This slight defect, previously observed in *M. thermolithotrophicus* (Belay et al., 1990) is less dramatic than diazotrophic conditions in which yields decreased by ≈58% (Figure 1C). When grown across a range of NaNO_3_ concentrations, growth was not observed below 1 mM NO_3_^−^ while the highest cell yield (Abs_600nm_) was observed at 10 mM, with an inhibitory effect at 100 mM (Supplementary Figure S1C). To monitor NO_3_^−^ utilization dynamics, growth was followed in the presence of 10 mM NaNO₃ over 25 h. NO_3_^−^ depletion closely mirrored biomass accumulation, with the culture entering the stationary phase around 14 h and NO_3_^−^ dropping below the detection threshold by 16 h (Figure 1B). Under these conditions, we could not detect the formation of NO_2_^−^ in the medium from the colorimetric test strips. Given the demonstrated NO_3_^−^-assimilating capacity of *M. infernus*, it was important to assess whether NO_3_^−^ could serve as a preferred nitrogen source when NH_4_^+^ is present, or whether diauxic growth would result from preferential utilization of NH_4_^+^. To address this, nitrogen source preference assays were performed by co-supplying 5 mM NaNO₃ and 5 mM NH₄Cl (Figure 1D). These experiments revealed that NH_4_^+^ was consistently consumed prior to NO_3_^−^. The pattern is consistent with the previous observation of lower cell yield (Abs_600nm_) in NO_3_^−^-grown cells than in NH_4_^+^-grown cells.

Although canonical NO_3_^−^ reductases are typically molybdenum dependent (Morozkina and Zvyagilskaya, 2007), *M. infernus* exhibited indistinguishable growth in both molybdenum-free and molybdenum-supplemented media (Figure 1C). As a control, we showed a defect in the growth of the molybdenum-deprived culture under N_2_-fixation, in which the molybdenum-dependent nitrogenase is essential (Maslać et al., 2025b). Nevertheless, we cannot exclude the possibility that a few contaminations of molybdenum in the medium might be enough to produce a molybdenum-containing NO_3_^−^ reductase that would be expressed at a lower level compared to the nitrogenase under diazotrophic conditions (Maslać et al., 2025b). It should also be noted that under fermenter conditions, the nitrogenase is expressed (Supplementary Figure S2A) and would absorb the available molybdenum. Taken together, a plausible explanation is that *M. infernus* NO_3_^−^ reductase functions via a tungsten-based pterin cofactor, allowing the molybdenum-independent growth as observed in *M. thermolithotrophicus* (Belay et al., 1990). The putative tungsten-dependent NO_3_^−^ reductase from *M. infernus* will not be the first of its kind since it has already been described in the hyperthermophile *Pyrobaculum aerophilum* (de Vries et al., 2010), and would rather be an adaptation to deep-sea microbes in which bioavailable molybdenum is scarce.

### The *in silico* predicted NO_3_^−^ reductase harbors all required molecular determinants for its activity

To dissect the pathway at the molecular level, we first focused on characterizing the NO_3_^−^ reductase. We consider this enzyme an appropriate marker based on the natural genetic mutation observed in *M. thermolithotrophicus* and its co-occurrent presence in *Methanococcales*, where NO_3_^−^ has been tested in the medium (Jeanthon et al., 1998; L'Haridon et al., 2003; Mehta and Baross, 2006). To characterize this enzyme, a native purification workflow was performed. Early chromatographic fractions displayed weak, albeit detectable NO_3_^−^-dependent benzyl-viologen oxidation, suggesting that the organism indeed harbors an active NO_3_^−^-reducing enzyme (Supplementary Figure S2B). However, enzyme activity rapidly diminished during the subsequent purification steps. Such activity loss might be due to the separation of redox partners or a metabolite activator (Lemaire et al., 2026; Müller et al., 2024), to alterations in the cofactor catalyst, or structural integrity. Because the loss of activity precluded native isolation, we turned to *in silico* analyses to verify that all molecular determinants conferring NO_3_^−^ reductase activity are present and to hypothesize about its origin.

**Figure 2:**
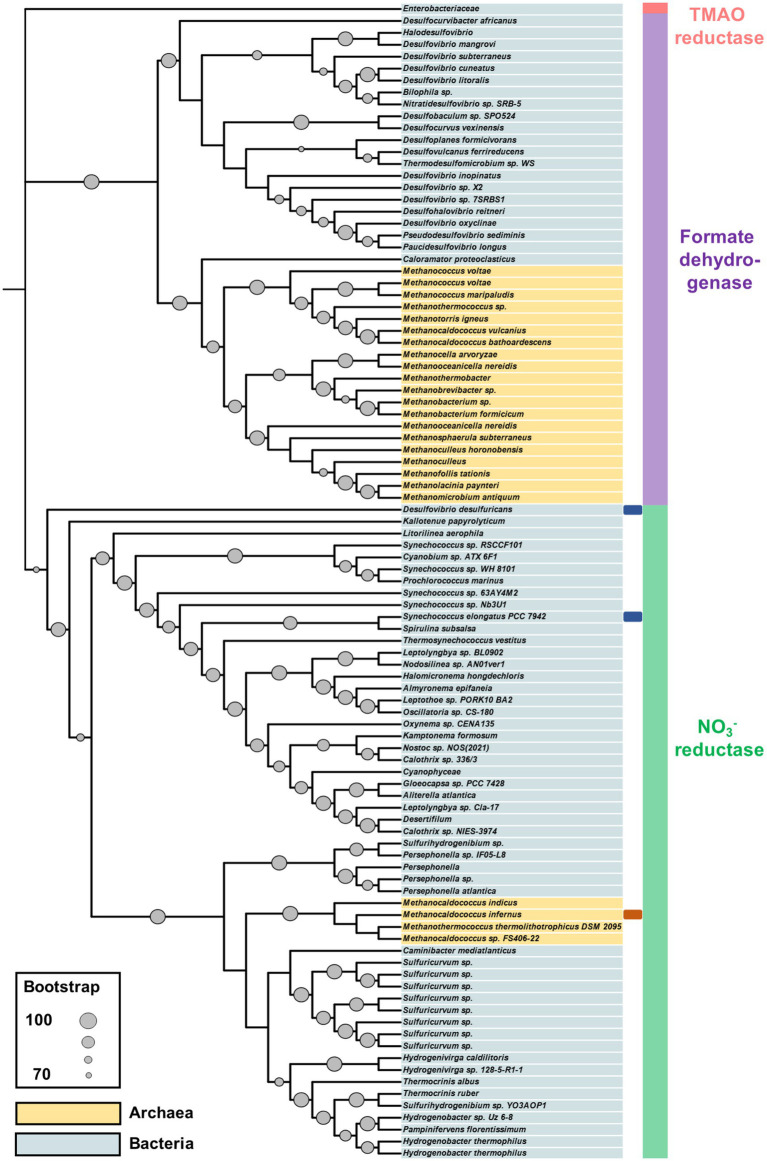
Phylogenetic tree of selected closest protein homolog sequences of *M. infernus* putative NO_3_^−^ reductase (brown square). Node statistics are represented by grey circles whose diameters depend on the node score (see the bootstrap legend). Bacteria representatives are represented in blue and archaea in yellow. TorA (Trimethylamine N-oxide reductase) from *Escherichia coli* was used as an outgroup. Accession numbers for sequences used in phylogenetic reconstruction can be found in Supplementary File S1. The tree was constructed in IQ-Tree3 (Wong et al., 2026) and visualized and annotated with ITOL (Letunic and Bork, 2024). Blue squares indicate NO_3_^−^ reductases that have previously been characterized and used in structural analysis (Supplementary Figure S3).

**Figure 3:**
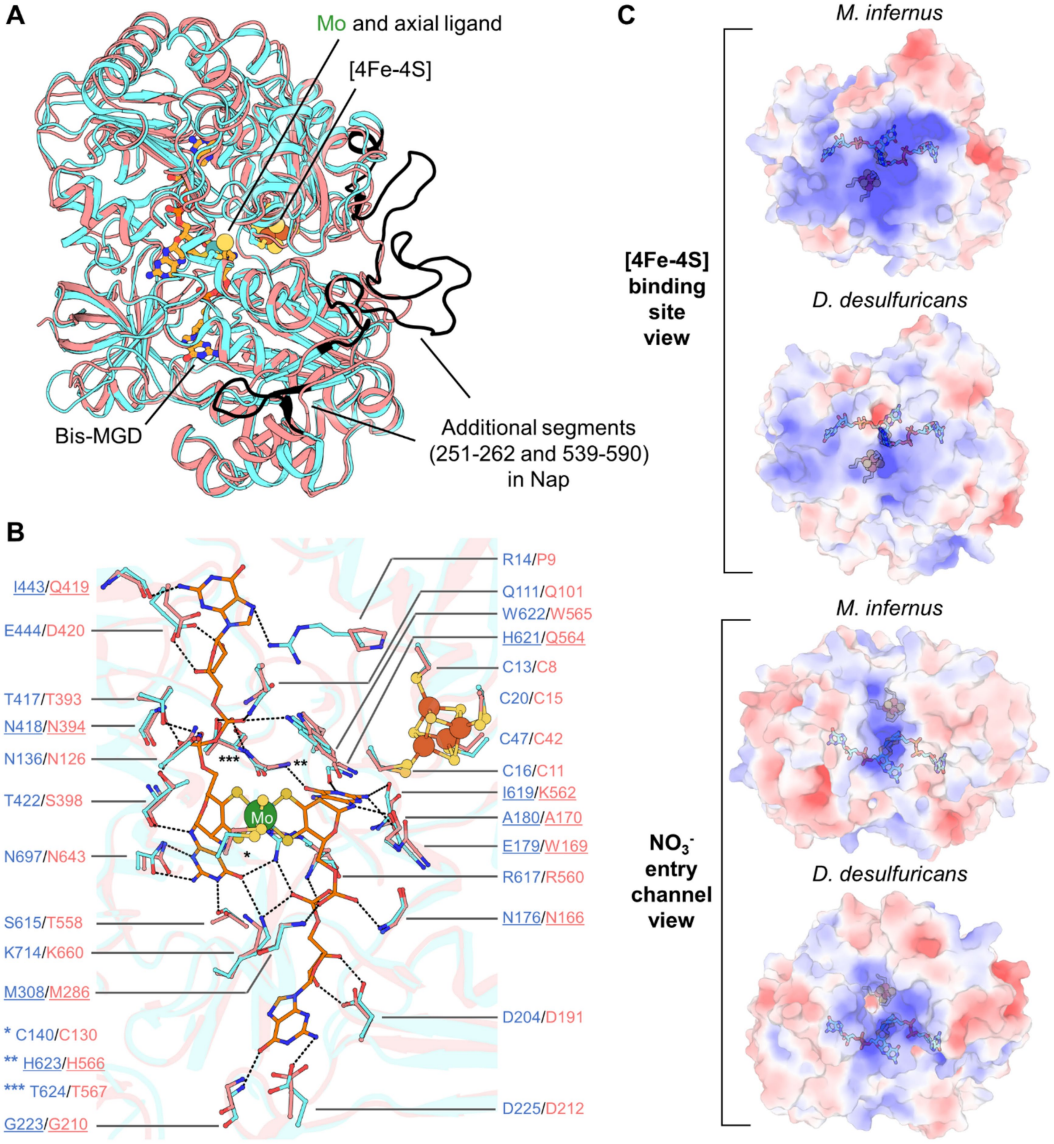
Comparison of the AlphaFold 3 (AF3) *M. infernus* putative NO_3_^−^ reductase model with *D. desulfuricans* Nap. **(A)** Superposition of AF3 *M. infernus* NO_3_^−^ reductase (light red) with Nap from *D. desulfuricans* (cyan, PDB 2JIO). The metalloclusters are shown as balls and sticks. **(B)** Close-up of the active site and the accessing [4Fe–4S] cluster for electron transfer with the same color coding as in **(A)**. Residues establishing covalent bonds or hydrogen bonds (black dashes) with the metallocofactors are shown in balls and sticks. The axial ligand, modelled here as a sulfido based from Najmudin et al. (2008). **(C)** Electrostatic charge profile comparison with a surface representation. Negative and positive patches are colored in red and blue, respectively. The NO_3_^−^ is accessible via a positively charged channel, and the [4Fe–4S] cluster positive surface patch is accessible to the electron donor partner. Oxygen, nitrogen, sulfur, iron, and molybdenum are colored in red, blue, yellow, orange, and green, respectively.

Phylogenetic inference placed this enzyme within the bacterial NO_3_^−^ reductases, showing an affiliation with a branch dominated by species from the *Aquificales* order, including many from the *Aquificaceae* family (Figure 2). Because many of these species share a similar deep-sea ecosystem with hyperthermophile *Methanococcales*, we propose that the NO_3_^−^ reductase has been acquired via horizontal gene transfer. However, none of these relatives has been characterized to date. To probe structural conservation, an AlphaFold3 model (Abramson et al., 2024) was generated, yielding consistently high confidence scores (overall pLDDT >90). Despite only 31% sequence identity with the homologue from *Desulfovibrio desulfuricans*, structural superposition demonstrated high similarity, with a root mean square deviation (RMSD) of 0.84 Å across 576 Cα atoms (Figure 3A). The predicted model exhibits the hallmark four-domain architecture of monomeric NO_3_^−^ reductase (Figure 3A and Supplementary Figure S3). Importantly, the canonical Cys-ligation for the [4Fe–4S] cluster and the molybdenum/tungsten center is fully conserved (Figure 3B). Most of the hydrogen bonding network required to coordinate the bis-molybdopterin guanine dinucleotide cofactor (bis-MGD) is present, which would suggest an identical metallocofactor in which molybdenum is replaced by tungsten.

Although the *M. infernus* model structurally resembles NapA, the functional implications of this similarity must be interpreted cautiously. Traditional descriptors such as “periplasmic,” “membrane-bound,” and “cytosolic,” historically applied to Nap, Nar, and Nas systems, often create ambiguity because these labels inconsistently overlap across different NO_3_^−^-reducing enzymes (Richardson, 2025). To avoid such pitfalls, we adopt the structural/bioenergetic nomenclature proposed by David Richardson, which classifies NO_3_^−^ reductases based on the Mo-ligand type (cysteine C-, or aspartate D-) and their orientation relative to the membrane potential side (positive P-, or negative N-side), forming the PC/PD/NC/ND scheme (Richardson, 2025). Within this framework, *Desulfovibrio* NapA is a PC-type enzyme localized to the periplasm and coordinated by a cysteine-ligated Mo. PC-type enzymes typically interact with NapC, a membrane-anchored multi-heme c-type cytochrome that oxidises quinol on the P-side of the membrane. By contrast, *M. infernus* lacks quinol-oxidising NapC equivalents and multi-heme c-type cytochromes, essential components required for a canonical PC-type NO_3_^−^ reductase. Accordingly, while the signal peptide and periplasm localization are predicted for the *Desulfovibrio* homologue (analyzed through DeepLocPro-1.0 and SignalP-6.0 server from the DTU Health Tech service, https://services.healthtech.dtu.dk/services/; Nielsen, 2025), the sequence from *M. infernus* is predicted to be without a signal peptide and cytosolic. Therefore, the NO_3_^−^ reductase of *M. infernus* aligns unambiguously with NC-type enzymes, with a cytosolic localization that would infer a different type of electron donor for the reaction. Unlike some bacterial NC-type enzymes, which contain extended polypeptides that bind additional [2Fe–2S] ferredoxin domains, or intrinsic NAD(P)H-oxidising domains, the *M. infernus* enzyme is simplified to the extreme, resembling the NC-type NO_3_^−^ reductase of *Synechococcus elongatus* or *Klebsiella pneumoniae* (Rubio et al., 1996; Cabrera et al., 2016; Lin et al., 1993; Lin et al., 1994). Electrostatic surface analysis of the AlphaFold3 model reveals a pronounced positive region surrounding the [4Fe–4S] cluster (Figure 3C), consistent with docking of an external ferredoxin-like partner analogous to cyanobacterial *S. elongatus* with a [2Fe-2S] cluster containing ferredoxin (Rubio et al., 1996; Srivastava et al., 2017) or the tungstopterin-dependent acetaldehyde-ferredoxin oxidoreductase with its 2x[4Fe–4S] cluster containing ferredoxin (Lemaire et al., 2026). Comparative electrochemical studies show that *S. elongatus* NC-type reductase operates at substantially lower redox potentials than its respiratory counterparts, consistent with the hydrogenotrophic cellular environment of *M. infernus* (Hirano et al., 2013). This configuration reflects the anabolic nature of NC-type NO_3_^−^ reductases, which couple NO_3_^−^ reduction to low-potential electrons derived from catabolic processes rather than to the high-potential quinols used by respiratory PC-, PD-, or ND-type enzymes (Simon et al., 2008).

To summarize, following our *in silico* analyses, we propose that the NO_3_^−^ reductase of *M. infernus* is a cytosolic Nas most likely relying on a 2x[4Fe–4S] cluster ferredoxin as an electron donor.

### An uncanonical NO_3_^−^ transporter with a plausible symporter function

**Figure 4:**
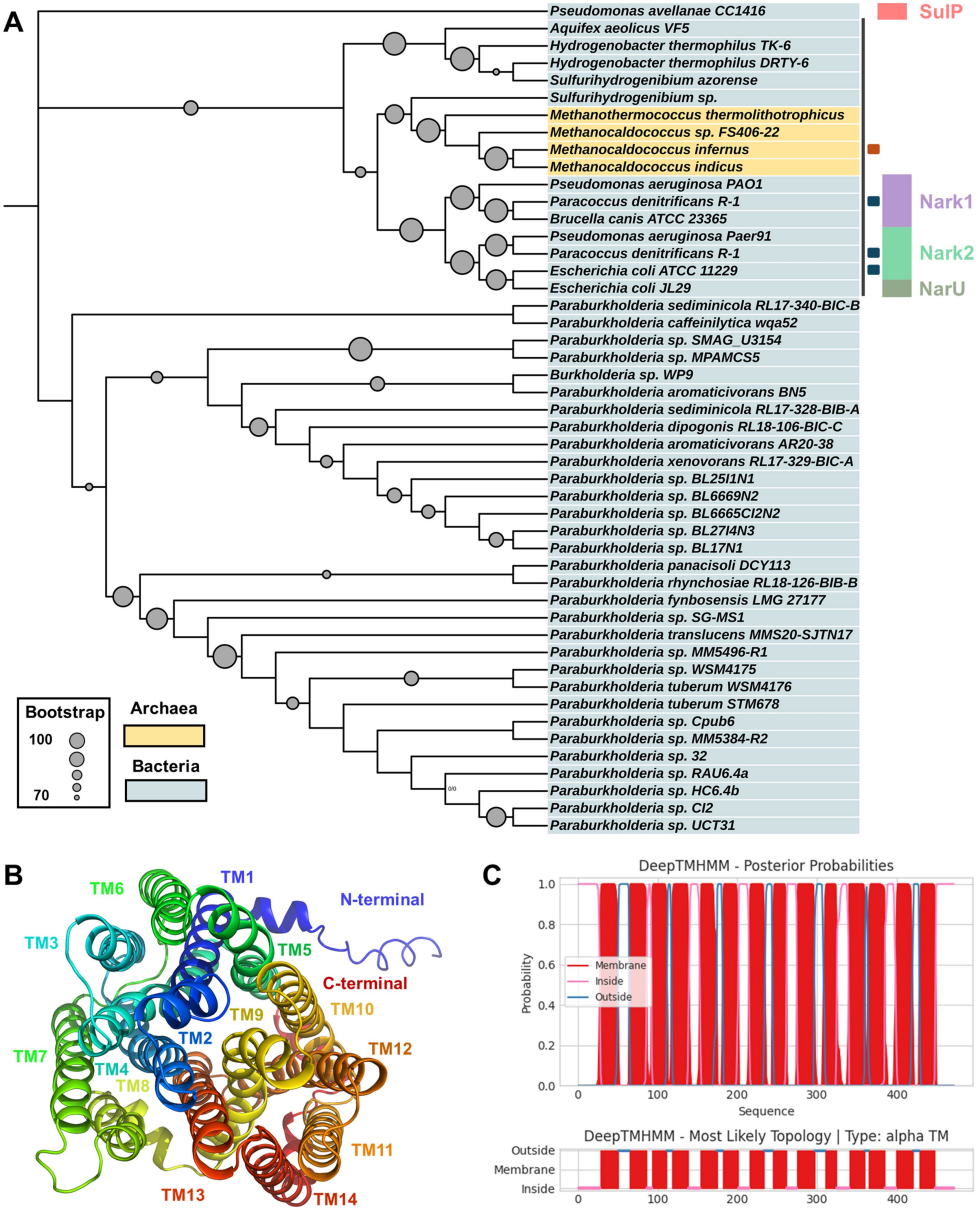
**(A)** Phylogenetic tree of selected closest protein homolog sequences of *M. infernus* putative NO_3_^−^ transporter (brown square). Homologues from *P. denitrificans* and *E. coli* are highlighted with a blue square. SulP from *P. avellanae* was used as an outgroup. Accession numbers are accessible in Supplementary File S1. The tree was constructed in IQ-Tree3 (Wong et al., 2026) and visualized and annotated with ITOL (Letunic and Bork, 2024). **(B)** Outside view of the AF3 model from *M. infernus* putative NO_3_^−^ transporter, including its transmembrane segments (TM). The cartoon is colored from the N- (blue) to the C-terminal (red). **(C)** Topology prediction of *M. infernus* NO_3_^−^ transporter by the DeepTMHMM server (Hallgren et al., 2022).

The *in silico* prediction of a cytosolic NO_3_^−^-reductase in *M. infernus* would imply an intracellular active transportation of NO_3_^−^. For this, the gene encoding a putative NO_3_^−^ transporter would fill this function. Here again, phylogenetic analyses indicate that within the *Aquificaceae* family, close homologues encode the NO_3_^−^ transporter concomitantly with NO_3_^−^ reductase (Supplementary Figure S1A). We also found close homologues (with ≈50% sequence identity) belonging to the *Burkholderiaceae* family, including many members from the *Paraburkholderia* genus (Figure 4A).

**Figure 5:**
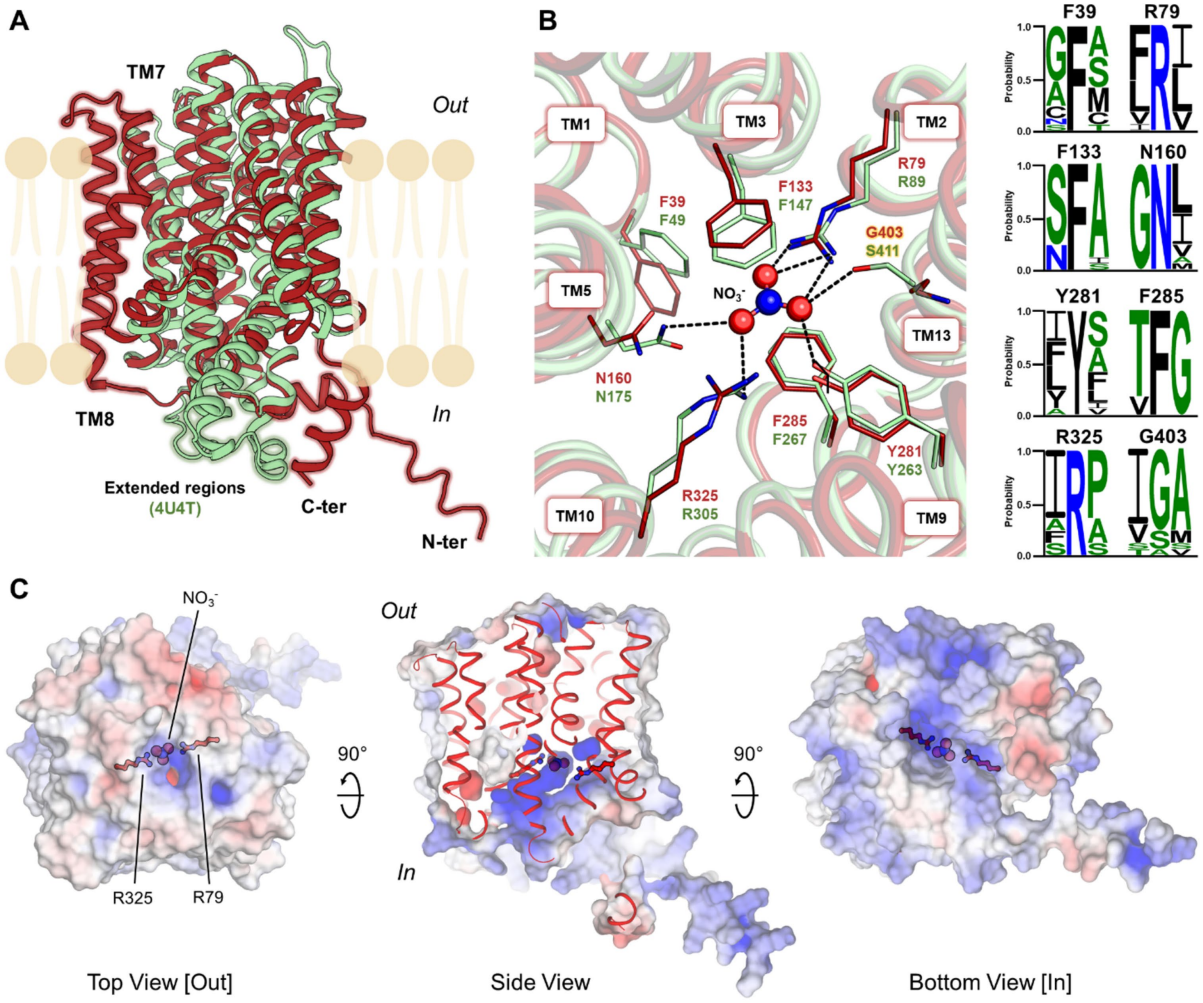
Comparison of the AF3 *M. infernus* putative NO_3_^−^ transporter model with *E. coli* NarK. **(A)** Superposition of AF3 *M. infernus* NO_3_^−^ reductase (red) with 4U4T from *E. coli* (light green). The extended region 380–401 in 4U4T, not existing in *M. infernus*, is highlighted in glowing green, while the extended N- and C- terminal regions and the 2 additional transmembrane helices (TM7 and TM8, see Supplementary Figure S4) from *M. infernus* are highlighted in glowing red. Topology was accessed through the DeepTMHMM web server (Hallgren et al., 2022) (see Figure 4C). **(B)** Close-up of the NO_3_^−^ binding site, with surrounding residues shown in sticks. The conservation score was obtained through the WebLogo server using the alignment of 16 NO_3_^−^ reductase sequences (Supplementary File S1). **(C)** Electrostatic charge profile comparison with a transparent surface representation. Negative and positive patches are colored in red and blue, respectively. The two conserved arginine and the NO_3_^−^ (the latter being modelled from PDB 4U4T) are displayed.

The AlphaFold3 model (Abramson et al., 2024) of the *M. infernus* transporter reveals structural features characteristic of NarK-family NO_3_^−^ transporters (Figure 4B,C,5A). NarK transporters can be classified into two main subgroups (Moir and Wood, 2001). NarK2-type transporters generally function as NO_3_^−^/NO_2_^−^ antiporters, exchanging intracellular NO_2_^−^ for extracellular NO_3_^−^, a mechanism suited to respiratory NO_3_^−^ reduction (Giffin et al., 2012; Clegg et al., 2002). By contrast, NarK1-type proteins are associated with NO_3_^−^/H^+^ symport, enabling direct NO_3_^−^ uptake for anabolic assimilation without NO_2_^−^ export (Goddard et al., 2017; Goddard et al., 2008; Gates et al., 2011). Here, phylogeny analyses place the *M. infernus* transporter firmly within a subclade of NarK2 subfamily (Figure 4A). Compared with the *E. coli* NarK2 representative, the *M. infernus* model showed a conserved NO_3_^−^-binding region that includes the conserved arginine-based N-oxyanion pocket at the interface of several transmembrane helices, creating an electrostatically favorable environment for NO_3_^−^ coordination (Figures 5B,C). Still, two important structural differences exist between the two models: a deletion of a segment between transmembrane helices 10 and 11 (residues 380–401 in *E. coli*), and an insertion of two additional transmembrane helix segments located between helices 7 and 8 in the *E. coli* homologue (Figures 5A,B and Supplementary Figure S4). These differences are unlikely to reflect adaptation to hyperthermophilicity or to the archaeal membrane environment, since very similar protein homologues exist in mesophilic bacteria (i.e., *Paraburkholderia aromaticivorans* NarK with 51.1% identity and 93% coverage). They could rather provoke a switch in the mechanism, turning the NO_3_^−^/NO_2_^−^ antiporter to a NO_3_^−^/ion symporter in which the ion transported could be protons or even Na^+^ (Yan et al., 2013). This hypothesis is supported by physiological evidence in which (i) NO_2_^−^ does not accumulate in the medium; (ii) the possible usage of the proton motive force to pump NO_3_^−^ into the cytoplasm actively would explain the decrease in biomass yield (Abs_600nm_). Given the physiological context, NO_2_^−^ should remain in the cytoplasm, where it can be catabolized into NH_4_^+^ for efficient anabolic assimilation.

### The NO_2_^−^ reductase step is fueled through F_420_H_2_-oxidation

Following down the path of NO_3_^−^-reduction, the highly toxic intermediate NO_2_^−^ will be generated. It must be immediately converted to NH_4_^+^ by a dedicated NO_2_^−^ reductase. In bacterial homologues, a NO_2_^−^ reductase is either directly located in the operon (i.e., *Persephonella atlantica*, Supplementary Figure S1A) or elsewhere in the genome. These genes encode a predicted large polypeptide composed of an N-terminal NAD(P)H-oxidase fused to a NO_2_^−^ reductase (i.e., *Aquifex aeolicus*).

**Figure 6:**
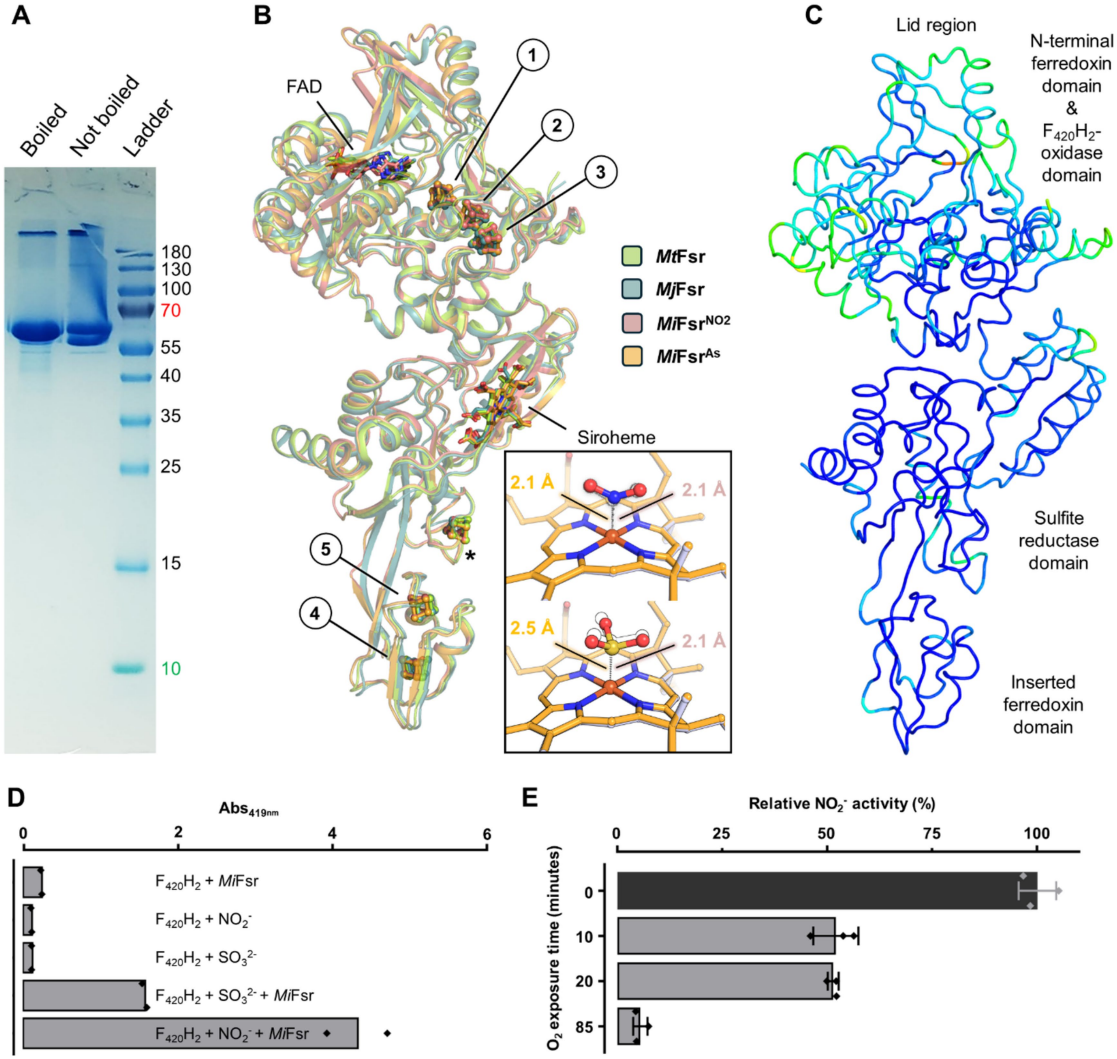
**(A)** SDS-PAGE profile of purified *Mi*Fsr. The sample was boiled for 3 min with the denaturing buffer or left standing. **(B)** Overall structural comparison between both *Mi*Fsr structures and their homologues is shown as a cartoon representation. The [4Fe−4S] cluster indicated by a star covalently binds the siroheme from the dimeric Fsr. A close-up of the active site of the NO_2_^−^ reductase is shown, comparing the position of NO_2_^−^ and SO_3_^2−^ between *Mi*Fsr^NO2^ and *Mi*Fsr^As^ structures. The slight elongation of the S-Fe distance between *Mi*Fsr^NO2^ and *Mi*Fsr^As^ might result from a radiation damage effect that over-reduces the heme (Gorel et al., 2025; Pfanzagl et al., 2020). Ligands are displayed as balls and sticks. **(C)** B-factor profile of *Mi*Fsr^NO2^ structure, highlighting more dynamics in the F_420_H_2_-oxidase domain as previously seen in Fsr homologues (Jespersen et al., 2023). **(D)** Absorbance 419 nm recorded after 88 s of reaction at 65 °C, confirming F_420_-dependent *Mi*Fsr activity using NO_2_^−^ or SO_3_^2−^ as substrates (see, Supplementary File S1: Figure 6). Experiments have been performed in duplicate. **(E)** Decrease of NO_2_^−^ reductase activity of purified MiFsr upon O_2_ exposition. Reduced benzylviologen was used as an electron donor for the reaction. Experiments have been performed in triplicate. For **(D,E)**, see the Methods section for the detailed protocol.

Because the enzyme in *M. infernus* must detoxify the poisonous NO_2_^−^, it was expected to exhibit a high turnover rate, facilitating its detection over the native anaerobic purification. Indeed, compared to NO_3_^−^ reductase activity, which was lost after the first chromatography step, benzyl-viologen-dependent NO_2_^−^ reductase activity could be tracked throughout the whole purification (see Methods). Because of its molecular weight, intrinsic black color (due to its high content of (metallo)cofactors), shift on denaturing gel between boiled versus unboiled sample (previously observed in our laboratory for *Mj*Fsr, data not shown, Figure 6A), and its known NO_2_^−^ reductase activity (Heryakusuma et al., 2023), the identity of the purified protein was presumed to be the F₄₂₀-dependent SO_3_^2−^ reductase (Fsr) (Jespersen et al., 2023). Therefore, the cofactor FAD was supplied during anaerobic crystallization. Following acquisition of the X-ray diffraction data, we used the *Methanocaldococcus jannaschii* Fsr model as a template for molecular replacement and experimentally confirmed that the NO_2_^−^ reductase was indeed Fsr.

Genomic analyses of *Methanococcales* harboring the *narK-nas* operon corroborate this finding, because the only enzyme encoded in the genome with known NO_2_^−^ reductase activity is Fsr. While *M. thermolithotrophicus*, *M. indicus*, and *M.* sp. FS406-22 harbor an *fsr* gene directly part of the *narK-nas* operon (Figure 1A and Supplementary Figure S1A), the one from *M. infernus* is located at a different position. It must also be noted that *M. thermolithotrophicus* and *M.* sp. FS406-22 contain an additional *fsr* isoform, which has been shown to serve for SO_3_^2−^ detoxification in *M. thermolithotrophicus* (Supplementary Figure S1B) (Jespersen et al., 2023).

**Figure 7:**
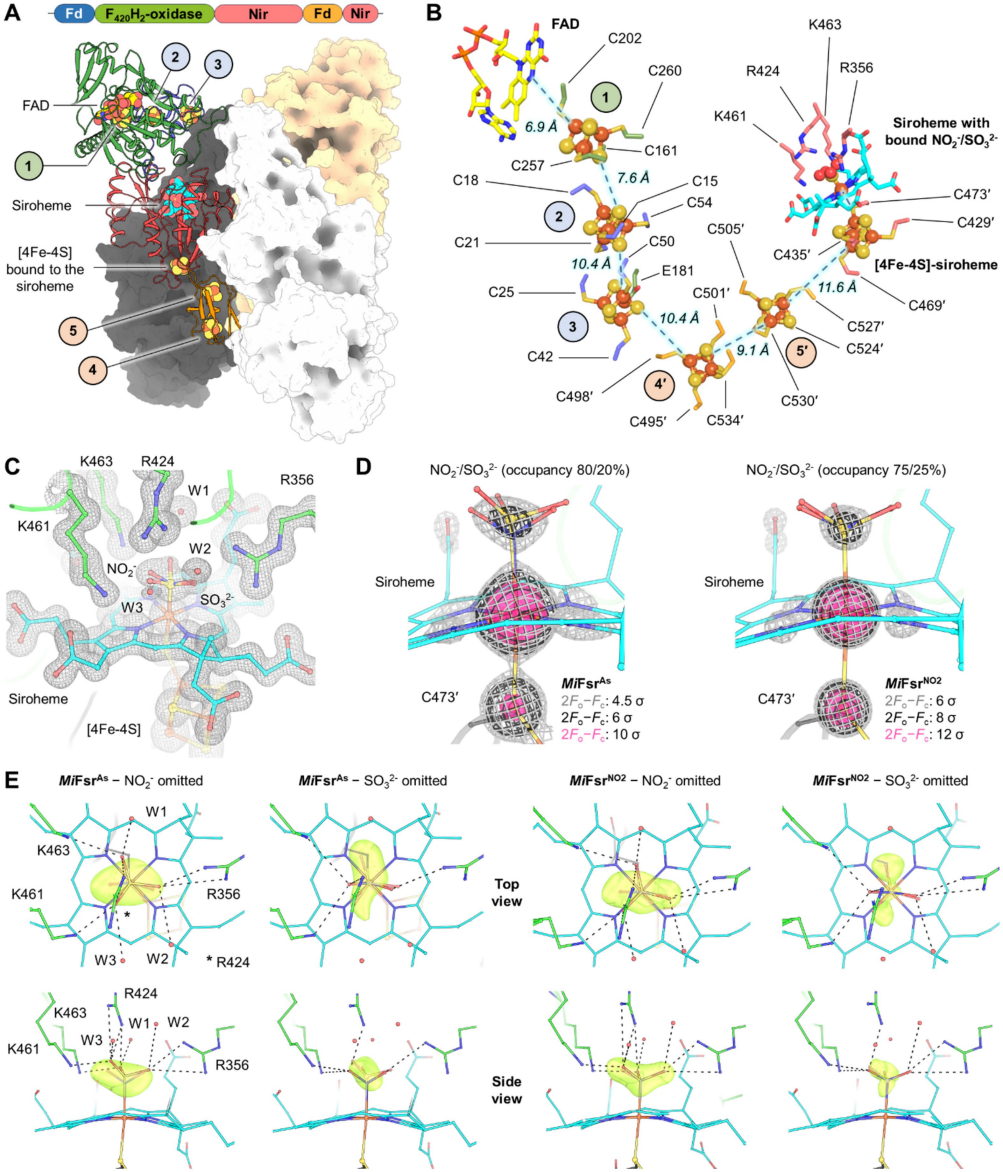
Fsr from *M. infernus* trapped with NO_2_^−^. **(A)** Overall view of *Mi*Fsr^NO2^ shown as a surface with one monomer in cartoon and ligands as balls. The top display illustrates Fsr organization with ferredoxin (Fd) and NO_2_^−^ reductase (Nir) domains. **(B)** Electron path from the FAD to the siroheme with the same color coding as in **(A)**. Primed numbers correspond to residues and clusters from the second protomer. **(C)** Close up of *Mi*Fsr^NO2^ active site with ligands and surrounding residues as sticks. The 2*F*_o_ − *F*_c_ map is contoured at 1-*σ*. **(D)** Close up of the axial ligand species bound to the siroheme contoured to different σ, revealing a partial occupancy of a SO_3_^2−^ species witnessed by the remaining signal (i.e., not compatible with a nitrogen). **(E)** Omit maps for NO_2_^−^ or SO_3_^2−^ and their coordination in both Fsr structures. The *F*_o_ − *F*_c_ resulting from the absence of NO_2_^−^ or SO_3_^2−^ is contoured to 7-σ and 5-σ, respectively.

We refined the crystal structures of *Mi*Fsr in its as-isolated form (*Mi*Fsr^As^) and after NO_2_^−^ soaking (*Mi*Fsr^NO₂^) to 1.58 Å and 1.21 Å, respectively (Table 1). As the two structures superimpose almost perfectly (RMSD of 0.14 Å for 623 Cα atoms aligned), *Mi*Fsr^NO₂^ served as the basis for detailed structural and mechanistic analysis (Figure 6B). *Mi*Fsr adopts the exact organization previously described in Jespersen et al. (2023) (Figures 6B, 7A), in which the overall homotetramer with a butterfly-like shape is composed of a SO_3_^2−^/NO_2_^−^ reductase core flanked by two 2[4Fe–4S]-ferredoxin and the F_420_H_2_-oxidase modules. As previously observed, the most dynamic part is located in the F_420_H_2_-oxidase domain, which has been proposed to depend on lid motion to bind the F_420_ cofactor and facilitate efficient electron delivery to the FAD (Figure 6C). The redox centers, constituted of the FAD, 6x[4Fe–4S] clusters, and the siroheme, are coordinated in the same way as *Mj*Fsr and *Mt*Fsr, allowing an efficient electron flow from the FAD isoalloxazine to the siroheme catalytic centre (Figure 7B). Accordingly, as isolated *Mi*Fsr was able to oxidise F_420_H_2_ *in vitro* upon NO_2_^−^ or SO_3_^2−^ addition (Figure 6D) and rapidly suffered from O_2_-exposition, most probably due to the [4Fe–4S] cluster oxidation (Figure 6E). Siroheme coordination in *Mi*Fsr mirrors that observed in *Mj*Fsr and *Mt*Fsr, where positively charged residues from one protomer stabilize the porphyrin, while the partner protomer binds the adjacent [4Fe–4S] cluster, generating the composite catalytic site (Figure 7C and Supplementary Figures S5, S6). The main difference between the structures lies in the Fe-adduct of the siroheme. Whereas *Mt*Fsr and *Mj*Fsr, respectively, harbor a sulfide and a SO_3_^2−^, *Mi*Fsr exhibits a mixture tentatively modelled as a NO_2_^−^ and SO_3_^2−^. Both ligands were first modelled in the atomic-resolution structure *Mi*Fsr^NO₂^, accounting for the B-factors to estimate their occupancies, being ~75% NO_2_^−^ and ~25% SO_3_^2−^ (Figures 7C–E). Based on the observed omit map for SO_3_^2−^, it is possible that a partially reduced species co-exists (i.e., bound sulfonyl). Similar occupancies (80% NO_2_^−^ and ~20% SO_3_^2−^) observed in *Mi*Fsr^As^ structure confirmed that the NO_2_^−^ soaking did not displace the bound ligands. The only notable difference on the siroheme site is an elongation of the S-Fe distance between *Mi*Fsr^NO₂^ (2.1-Å distance) and *Mi*Fsr^As^ (2.5-Å distance), which most probably comes from an artificial reduction induced by X-ray radiation damage (Figure 6B). This highlights that NO_2_^−^ and SO_3_^2−^ are most likely endogenous ligands that were trapped during cell harvesting and cell lysis. The presence of SO_3_^2−^ might result from contamination of the sulfide stock used during cultivation or from oxidation by O_2_ traces.

As for *Mj*Fsr, the SO_3_^2−^-bound ligand is coordinated via three waters, and the conserved Arg356, Arg424, Lys461 and Lys463. The NO_2_^−^ is also coordinated by Arg356, Arg424, Lys461 and Lys463 (Supplementary Figure S6). Both arginines are located at similar positions in the plant NO_2_^−^ reductase (i.e., Arg109 and Arg179 in the structural homologue from Tobacco leaf), but the side chain of the homologous residue of Lys461 shifts towards the core of the protein. The relocation of this side chain cannot happen in Fsr because Lys463 already occupies a similar position, which is substituted by an asparagine in the plant homologue. The difference in lysine position might explain the slight difference in NO_2_^−^ position. However, it is not expected to negatively affect enzyme turnover. The latter argument is also pointed out by the fact that all *fsr* isoforms detected in the *narK-nas* containing *Methanococcales* systematically harbor the lysine instead of an asparagine (Supplementary Figure S5).

## Discussion

NO_3_^−^ is rarely a preferred nitrogen source for methanogenic archaea because it is transiently present in anoxic habitats where it is rapidly scavenged by high-affinity NO_3_^−^-respiring microbes, outcompeting methanogens for substrates such as hydrogen (Chidthaisong and Conrad, 2000) and further restricting the narrow window in which methanogens could benefit from NO_3_^−^ assimilation. Besides ecological competition, NO_3_^−^ and the intermediate NO_2_^−^ exert additional negative effects, as previously observed in cultures (Belay et al., 1990; Klüber and Conrad, 1998). As a result, adaptation to NO_3_^−^ assimilation by methanogens is a peculiar trait that must be confined to specific ecological settings where NO_3_^−^ is sporadically available while other nitrogen sources, such as ammonia, are exhausted. From our RefSeq-based analyses, we conclude that only three currently sequenced methanogens appear to have the complete sets of genes to support NO_3_^−^ utilization (*Methanocaldococcus indicus*, *Methanocaldococcus infernus*, and *Methanocaldococcus* sp. FS406-22), providing an opportunity to examine the molecular basis of the pathway. Based on physiology and genetic analyses, we further infer that *M. thermolithotrophicus* DSM 2095 (the same strain used in the studies by Belay in 1990 for NO_3_^−^ utilization), should have progressively lost its capacity to utilize NO_3_^−^ due to a stop codon in the NO_3_^−^ reductase gene identified in the genome sequence released in 2013 (NCBI reference sequence: NZ_AQXV01000018.1) and later on 2021 by our group (NCBI reference sequence: NZ_OX296583.1) (Maslać et al., 2022). This may result from repeated laboratory transfers under NO_3_^−^ deprived conditions and represents a striking example of how “tamed” microbes under laboratory conditions can lose their natural traits. Nevertheless, extending the analysis beyond RefSeq suggests that additional *Methanococcales* may retain the capacity for NO_3_^−^ utilization (e.g., *Methanothermococcus thermolithotrophicus* strain ST22 does not contain the internal stop codon in the NO_3_^−^ reductase gene, accession number: XMX99672.1). Consistent with this, *M. infernus* can grow in Duran bottles or in fermenters containing solely NO_3_^−^, with millimolar concentrations sufficient to sustain robust growth, as previously shown for NH_4_Cl in other *Methanococcales* (Belay et al., 1990; Maslać et al., 2022).

The current working hypothesis is to assimilate extracellular NO_3_^−^, *M. infernus* would rely on the following sequential steps: (i) NO_3_^−^ would be actively pumped in via the NarK symporter that could have evolved from a NarK2 NO_3_^−^/NO_2_^−^ antiporter; (ii) intracellular NO_3_^−^ is reduced by a Nas, which most likely rely on ferredoxin as an electron donor for the reaction; (iii) the reactive NO_2_^−^ is fully reduced to NH_4_^+^ by Fsr, consuming F_420_H_2_ to supply electrons; (iv) the glutamine synthetase branches NH_4_^+^ to glutamate by hydrolyzing ATP to form glutamine; (v) a putative F_420_-dependent glutamate synthase (Susanti and Mukhopadhyay, 2012) generates two glutamate from glutamine and 2-oxoglutarate; (vi) aminotransferases distribute the amino-group of the glutamate for anabolic reactions.

The extremely high exergonic process of NO_2_- reduction by Fsr (Δ*G*°′ = −457 kJ/mol) (Jespersen et al., 2023; Heryakusuma et al., 2022) has a dual function, nitrogen assimilation and NO_2_^−^ detoxification. It is particularly important as NO_2_^−^ is a highly reactive oxidant that strongly inhibits the methyl-coenzyme M reductase (Duin et al., 2016), leading to the collapse of methanogenesis and energy acquisition. Fsr NO_2_^−^ reductase activity has been reported several times *in vitro* (Jespersen et al., 2023; Heryakusuma et al., 2023), including in non-NO_3_^−^ utilizers, but the presented structure of the endogenous NO_2_^−^-Fsr complex underlined its physiological function *in vivo*. Since *M. infernus* harbors a single *fsr* gene copy in its genome, its Fsr would have a dual function for NO_2_^−^ and SO_3_^2−^ detoxification. Fsr dual functionality is consistent with evolutionary models proposing that modern SO_3_^2−^-reductases originated from an ancestral Sir/Nir fusion, and it is not unprecedented among methanogens (Jespersen et al., 2023; Heryakusuma et al., 2023; Susanti and Mukhopadhyay, 2012). In comparison, *M. thermolithotrophicus* or *M.* sp. FS406-22 harboring two isoforms might have a differentiation based on their expression rather than catalytic activity to respond independently to NO_2_^−^ or SO_3_^2−^ stress. It must be noted here that the Fsr isoform localized in *narK/nas* operon of *M. thermolithotrophicus* harbors a substitution of the Arg424 (*Mi*Fsr sequence) for a threonine, which would normally coordinate the NO_3_^−^ or SO_3_^2−^ (Supplementary Figure S5). Such a substitution might either be a specialization for NO_2_^−^ detoxification or result from a degeneration due to the loss of the ability to assimilate NO_3_^−^. Although the functional implications are unknown, it has been shown that the Fsr encoded in anaerobic methane-oxidizing archaea (FsrII from ANME) also harbors a substitution at that site, exchanging the arginine for a glycine, and in addition, the Arg356 (*Mi*Fsr sequence) for a Lysine (Heryakusuma et al., 2022). The recombinant expression of FsrII from ANME showed a F_420_H_2_-dependent NO_2_^−^ activity, but no SO_3_^2−^ utilization, and the authors proposed a specialization toward NO_2_^−^ utilization.

**Figure 8:**
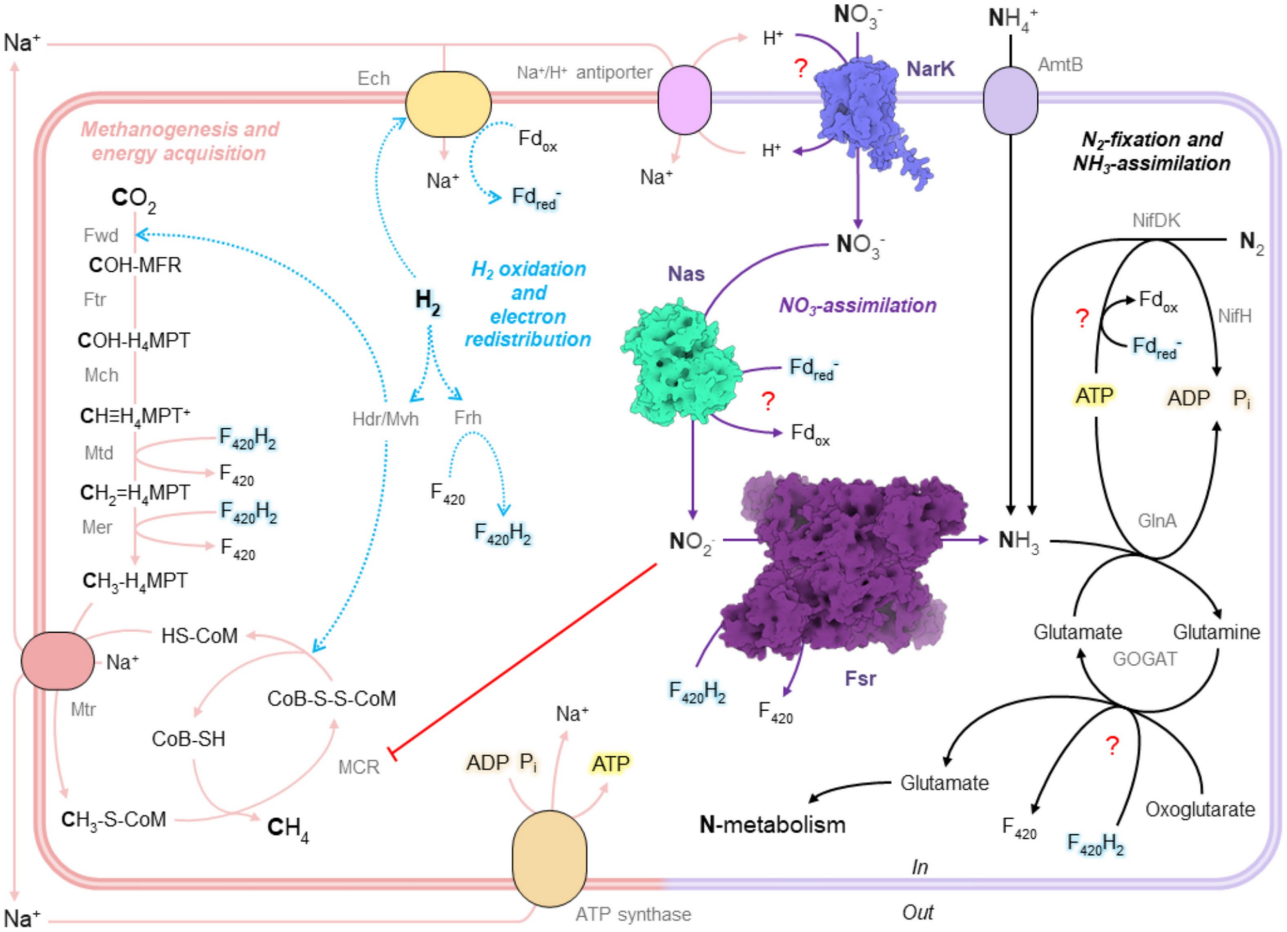
Proposed NO_3_^−^ reduction pathway in *M. infernus*. The presented NarK and Nas structures come from AF3 models, and Fsr is from the experimental model *Mi*Fsr^NO2^. The stoichiometry of the presented reactions and the water or protons (except for NarK) is not respected for clarity. MFR, H_4_MPT, CoM, CoB, Fd stand for Methanofuran, tetrahydromethanopterin, Coenzyme M, Coenzyme B, and Ferredoxin, respectively. NarK putative symporter might transport H^+^ or Na^+^.

Still, the energetic burden associated with NO_3_^−^ utilization reduces final biomass yields (Abs_600nm_) by ≈16% in *M. infernus* and ≈25% *M. thermolithotrophicus* (Belay et al., 1990). The rational explanation lies firstly in the mechanical energy required to pump NO_3_^−^ into the cytosol, which could depend on the ion gradient produced during methanogenesis (Figure 8). Secondly, if ferredoxin is the electron donor for NO_3_^−^ reduction, then Nas would indirectly consume the sodium gradient via the energy-converting hydrogenases (Ech). Thirdly, Fsr will consume part of the F_420_H_2_ required for methanogenesis and anabolic purposes. The latter would not affect growth under non-H_2_-limited conditions, but is expected to be problematic in natural environments where H_2_ concentration fluctuates. Nevertheless, the energy invested in using NO_3_^−^ remains reasonable compared to the toll imposed by the N_2_-fixation machinery that co-occurs in the four *narK-nas*-containing *Methanococcales*.

Despite our analyses on the pathway, several key questions remain unresolved. It is not yet known whether the NO_3_^−^ transporter functions as a symporter or antiporter, nor is the identity of the physiological redox partner supplying electrons to the Nas, which seems to be a tungsten-dependent version (Figure 1C). Using genetically tractable methanogenic hosts such as the hyperthermophile *M. jannaschii* (Susanti et al., 2019), which naturally reduce NO_2_^−^ to NH_4_^+^ through Fsr, could represent an excellent chassis to produce recombinant tagged NarK and Nas, proving that only these two genes are sufficient to confer NO_3_^−^ utilisation and enabling their biochemical characterisation.

## Conclusion

This work provides a molecular explanation of the NO_3_^−^-assimilation pathway used by hydrogenotrophic *Methanococcales*. The proposed metabolism would require the acquisition of the NO_3_^−^ transporter and NO_3_^−^ reductase, most probably inherited through lateral gene transfer from a member of the *Aquificaceae*, as suggested by phylogenetic analyses. Then, the recruitment of Fsr as the NO_2_^−^-reducing enzyme provides an efficient mechanism to couple F_420_H_2_-oxidation with NO_2_^−^ detoxification. Such integration of Fsr echoes the sulfate-assimilation pathway described in *M. thermolithotrophicus*, in which the methanogen hijacked parts of the machinery from sulfate reducers, remodeling enzymes and repurposing others to meet its metabolic needs (Jespersen and Wagner, 2023). Such adaptive versatility likely plays a pivotal role in enabling methanogens to capitalize on fluctuating resources in their natural habitats while minimizing energetic burden by integrating the pathway without interfering with energy-acquisition metabolism.

## Data Availability

The datasets used for the x-ray crystallography experiments are available in the NCBI Protein Database (see Table 1 for PDB ID codes). Additional data are available in the supplementary materials.
