# Supplementary material for: Nitrate reduction salvage pathway in Methanococcales
Source: Front Microbiol. 2026 Jul 6;17:1824787. doi: 10.3389/fmicb.2026.1824787 (PMC13381448; doi:10.3389/fmicb.2026.1824787)
Supplement: Supplementary file 1 [file supplementary_file_1.pdf]

Nitrate reduction salvage pathway in *Methanococcales*

Amelie Heidenreich, André G. Gouveia, and Tristan Wagner*

Supplementary material

This document contains Figures S1-S6

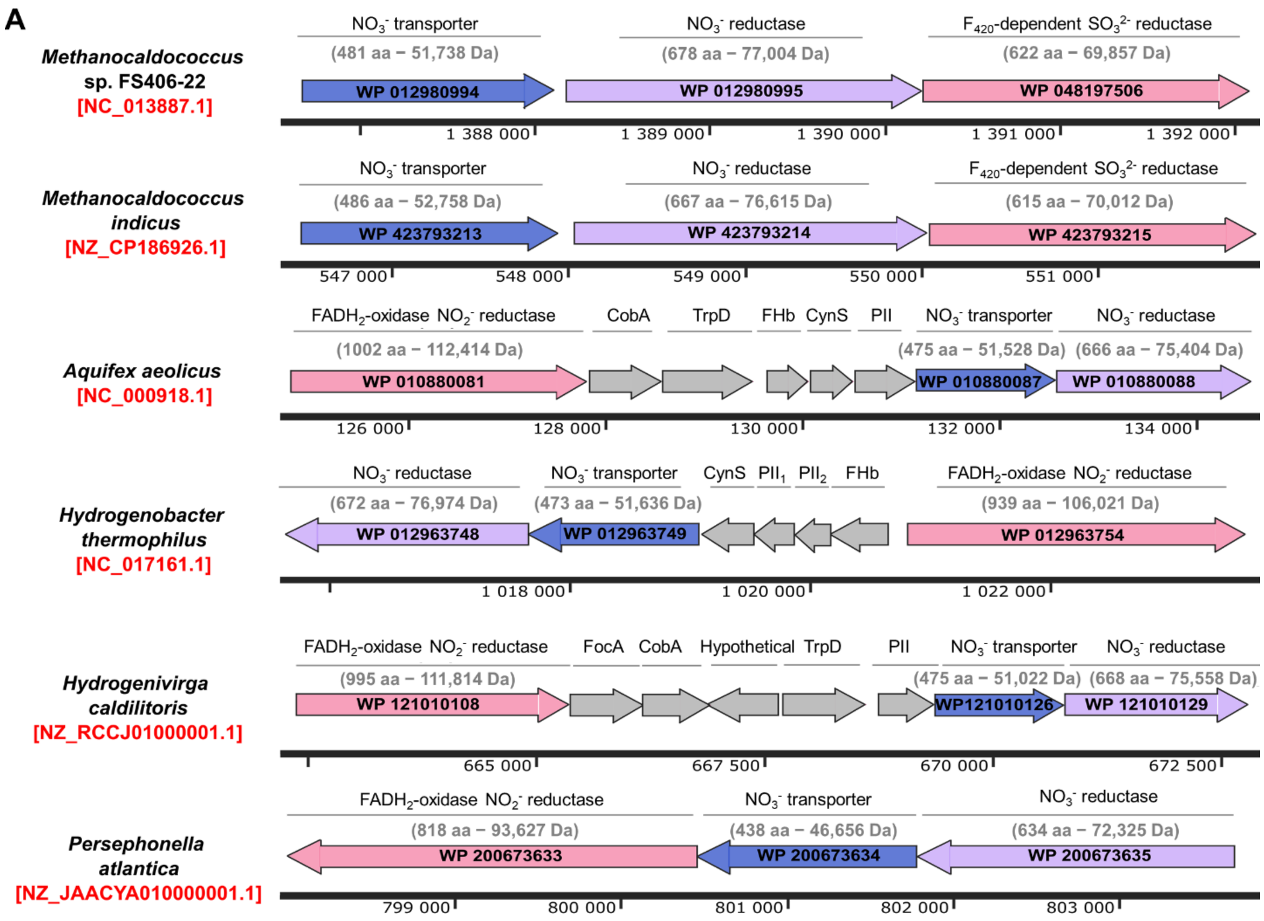

B

NO_3^- reductase

% sequence identity	*M. infernus* WP_013099975	*M. indicus* WP_423793214	*M.* FS406-22 WP_012980995	*M. thermo.* Gene_RS07920*
M. infernus	100	69.5	77.6	73.0
M. indicus		100	69.2	66.7
M. FS406-22			100	78.4

NO_3^- transporter

% sequence identity	*M. infernus* WP_013099974	*M. indicus* WP_423793213	*M.* FS406-22 WP_012980994	*M. thermo.* WP_018153296
M. infernus	100	80.0	81.0	76.9
M. indicus		100	77.5	73.9
M. FS406-22			100	83.4

F_{420}-dependent SO_3^{2-} reductase

% sequence identity	*M. infernus* WP_013100746	*M. indicus* WP_423793215	*M.* FS406-22 isoform 1 WP_012980875	*M. thermo.* isoform 1 WP_119720511	*M.* FS406-22 isoform 2 WP_048197506	*M. thermo.* isoform 2 WP_263315238
M. infernus	100	68.7	66.0	64.7	66.6	62.4
M. indicus		100	67.1	64.0	66.1	62.3
M. FS406-22 isoform 1			100	81.1	84.1	75.6
M. thermo. isoform 1				100	75.2	84.5
M. FS406-22 isoform 2						74.4

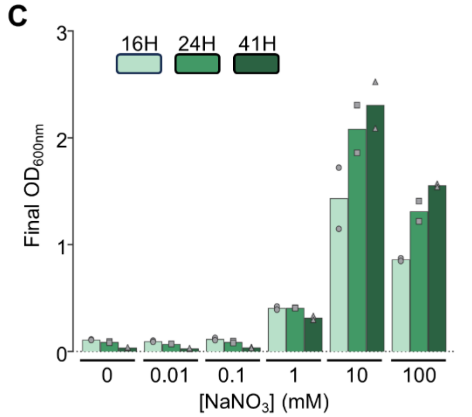

Fig. S1. A, Arrangement of the predicted operon for NO_3^- reduction in methanogens and selected bacteria. The NO_2^- reductase in bacteria appears to be an $FADH_2$-oxidase fused to the reductase, potentially using NAD(P)H as an electron donor. **B**, Sequence identities between close homologs for the NO_3^- reductase, transporter, and F_{420}-dependent SO_3^{2-} reductase. *M. thermo*. stands for *M. thermolithotrophicus*. **C**, OD_{600} of *M. infernus* cultures after 16, 24, and 41 h of incubation with different $NaNO_3$ concentrations (n=2).

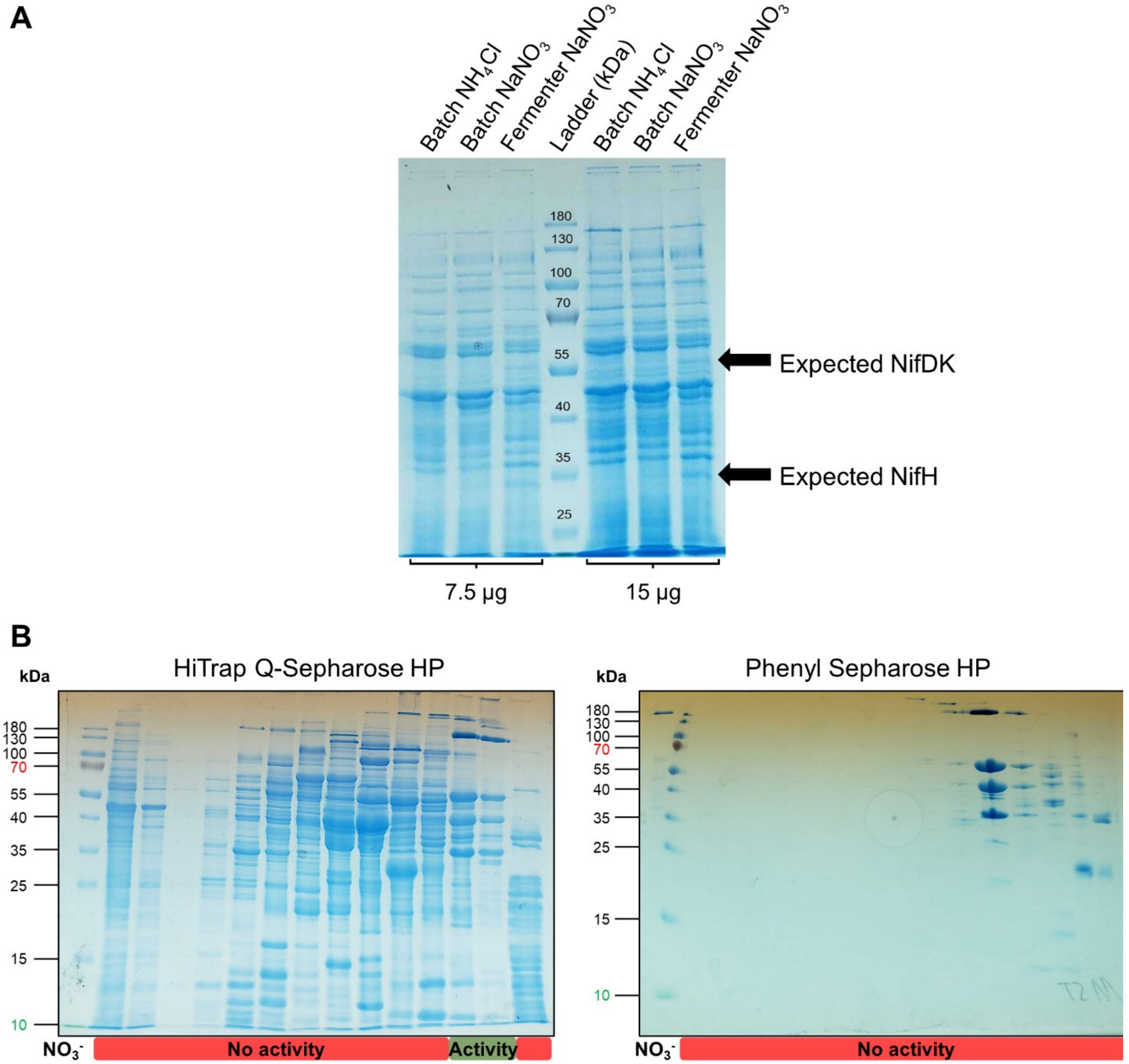

Fig. S2. **A**, Cell extract of *M. infernus* cultures loaded on SDS-PAGE. The cell extracts were obtained from NH_4Cl-grown cells and $NaNO_3$-grown cells in bottles (i.e., batch) or $NaNO_3$-grown cells in a fermenter. The 7.5 and 15 µg indicate the amount of protein loaded onto SDS-PAGE. The expected bands for the nitrogenase, constituted of NifDK and NifH, are highlighted and have been studied in Maslać et al. 2025 (1). **B**, Benzylviologen-based NO_3^- reductase activity in different protein fractions from anionic exchange chromatography (left) followed by hydrophobic interaction (right). The fractions containing a weak NO_3^- reductase activity detected on the Q-Sepharose fraction were pooled and injected on the Phenyl Sepharose, in which the activity could not be detected anymore.

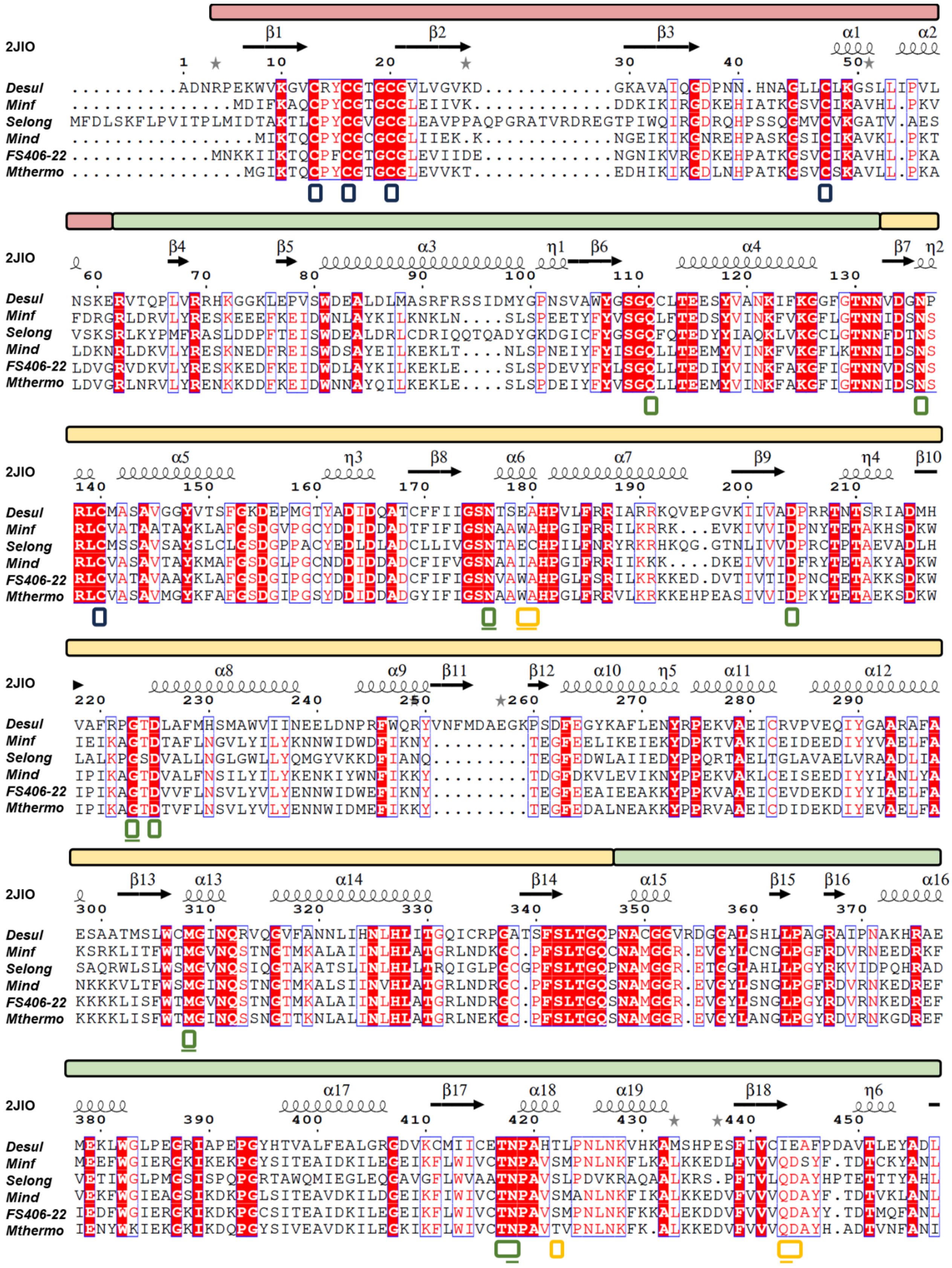

Fig. S3. See next page for the legend.

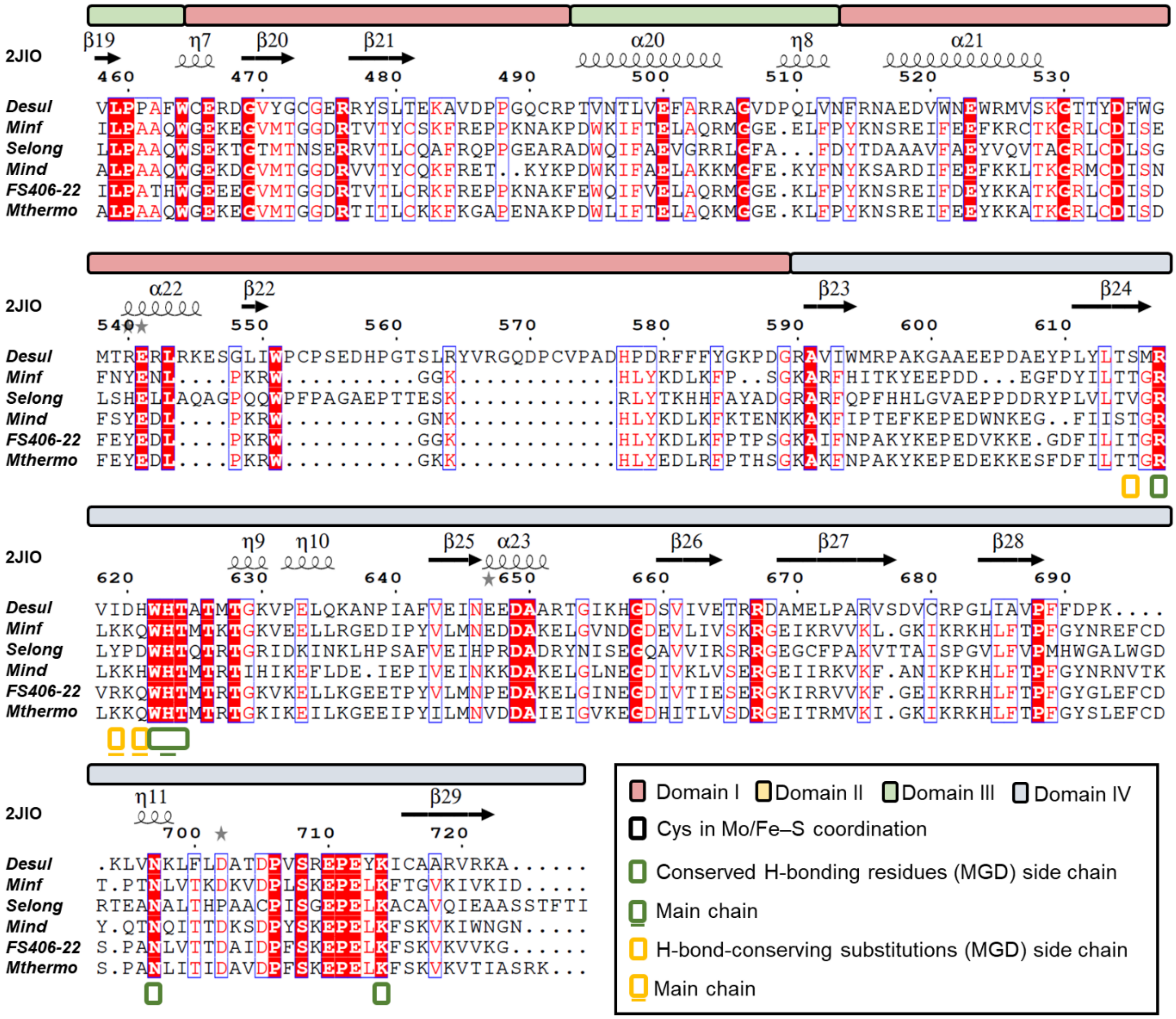

Fig. S3. Sequence conservation between the NO_3^- reductase from *D. desulfuricans* (PDB 2JIO), *Synechococcus elongatus* PCC 7942 (*Selong*), and *Methanococcales*. The secondary structure from *D. desulfuricans* is superposed on the sequence alignment. The four-domain separation is based on Dias et al. (2). The figure was generated by the Espript server (3). *Desul*, *Minf*, *Selong*, *Mind*, *FS406-22*, and *Mthermo* stand for *Desulfovibrio desulfuricans*, *Methanocaldococcus infernus*, *Synechococcus elongatus* PCC 7942, *Methanocaldococcus indicus*, *Methanocaldococcus* FS406-22, and *Methanothermococcus thermolithotrophicus*.

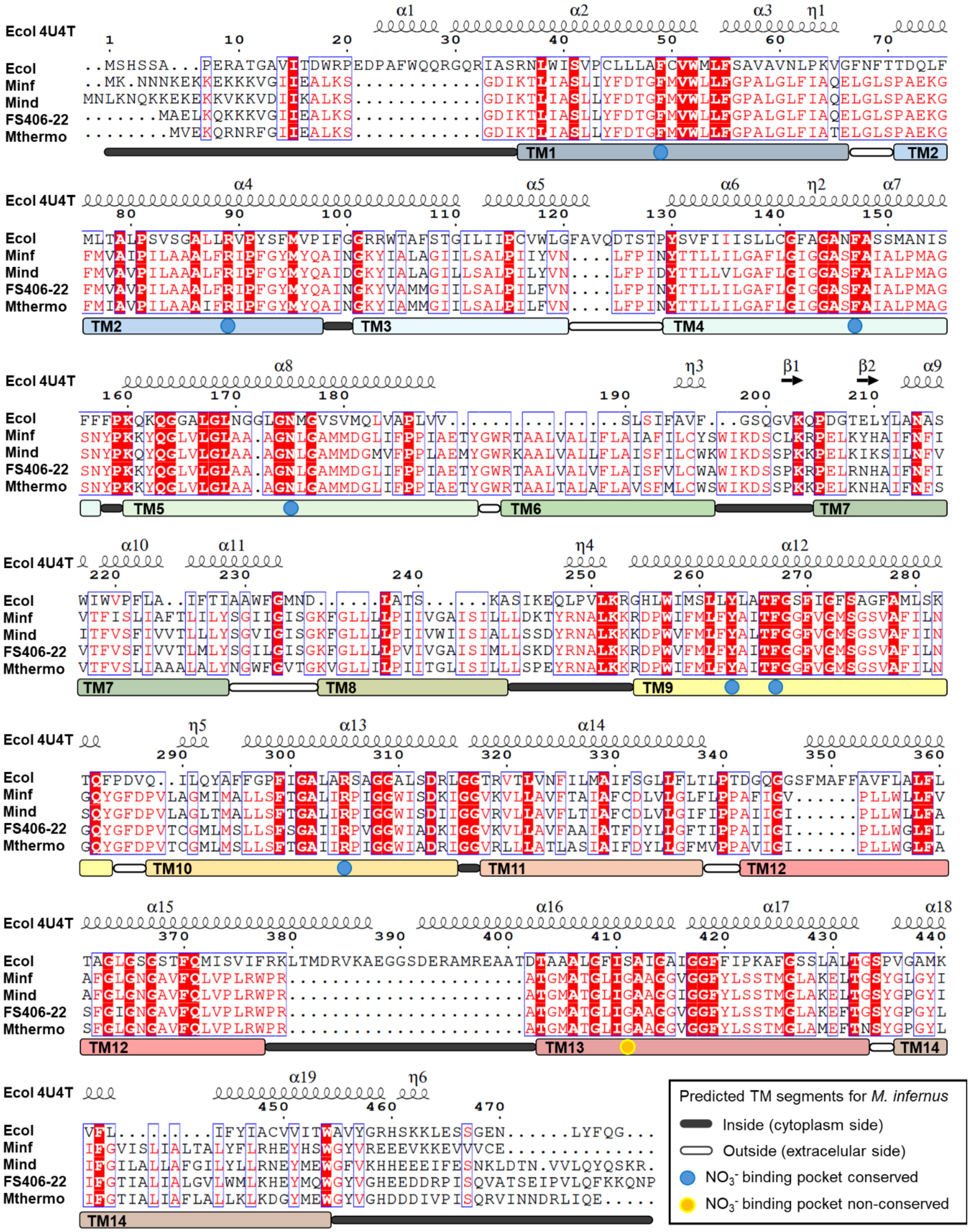

Fig. S4. Sequence conservation between the NO_3^- transporter from *E. coli* (Ecol, PDB 4U4T) and *Methanococcales*. The secondary structure of *E. coli* is superimposed on the sequence alignment and was generated using the ESPript server (3). Microbe abbreviations are the same as in Fig. S4. *E. coli* sequence from 189 to 247 does not match the *Methanococcales* sequence, which contains two additional TMs. Predicted TM segments of *M. infernus* transporter are shown below the sequence alignment.

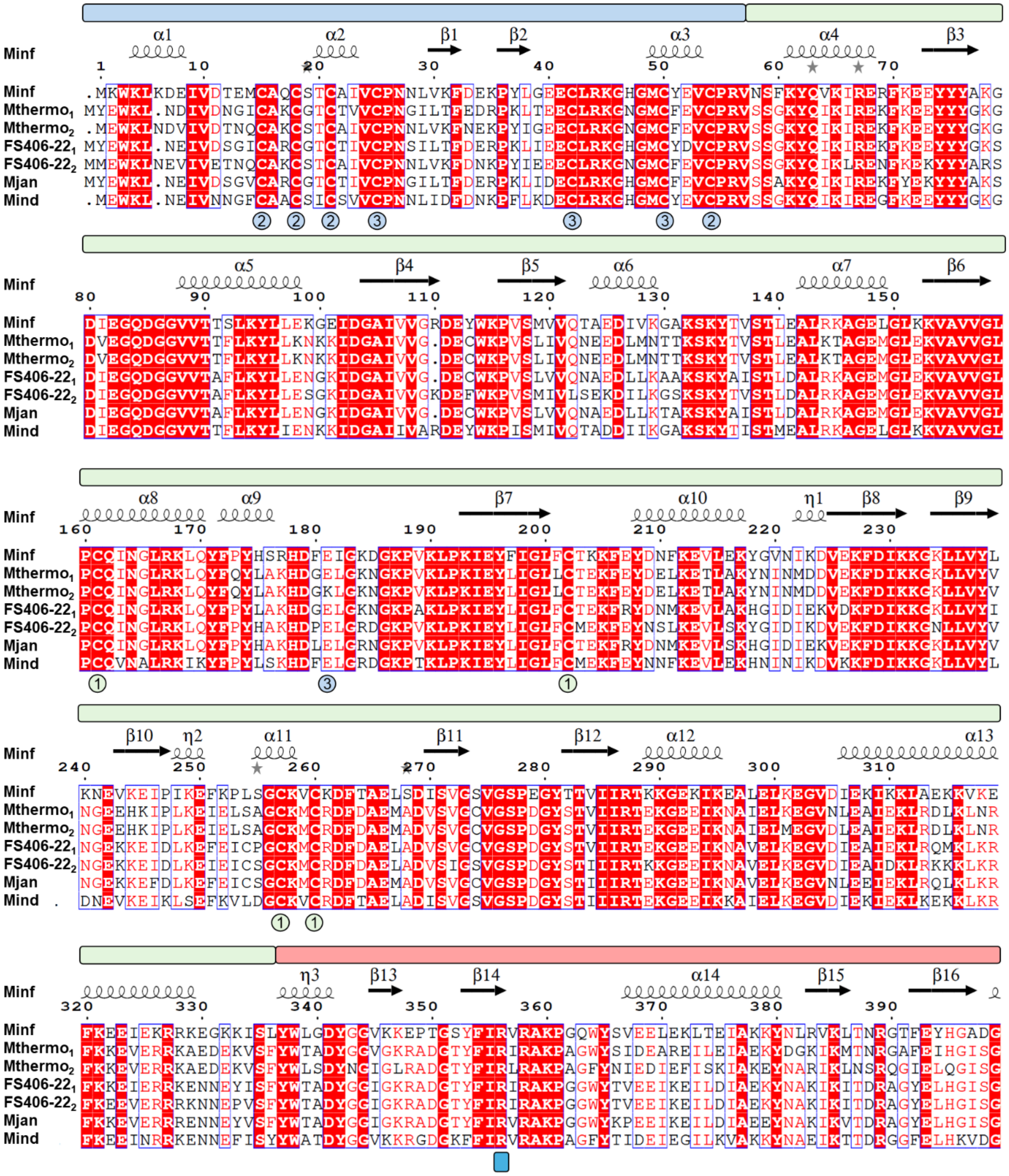

Fig. S5. See next page for the legend.

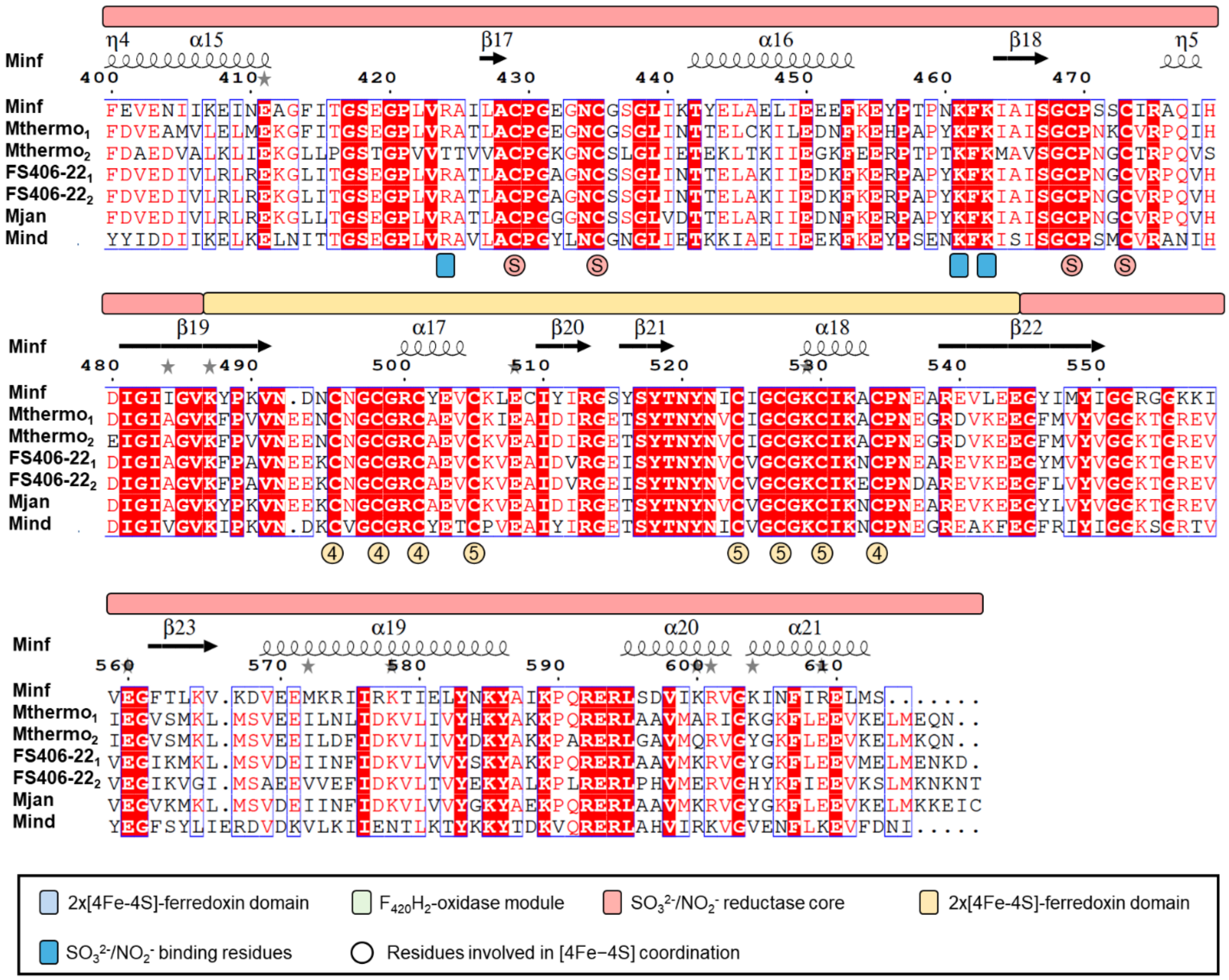

Fig. S5. Sequence conservation between Fsr from *M. infernus* and *Methanococcales*. The secondary structure from *M. infernus* is superposed on the sequence alignment. The four-domain separation is based on Jespersen et al. 2023 (4). The figure was generated by the ESPript server (3). For the residues binding the [4Fe–4S], numbers are followed by the same nomenclature as Fig. 7, and “S” indicates the cluster covalently bound to the siroheme. Minf, $Mthermo_1$, $Mthermo_2$, FS406-22_1, FS406-22_2, Mjan, and Mind stand for *Methanocaldococcus infernus*, *Methanothermococcus thermolithotrophicus* Fsr isoform 1, *M. thermolithotrophicus* Fsr isoform 2, *Methanocaldococcus Sp.* FS406-22 Fsr isoform 1, *Methanocaldococcus Sp.* FS406-22 Fsr isoform 2, *Methanocaldococcus jannaschii* and *Methanocaldococcus indicus*.

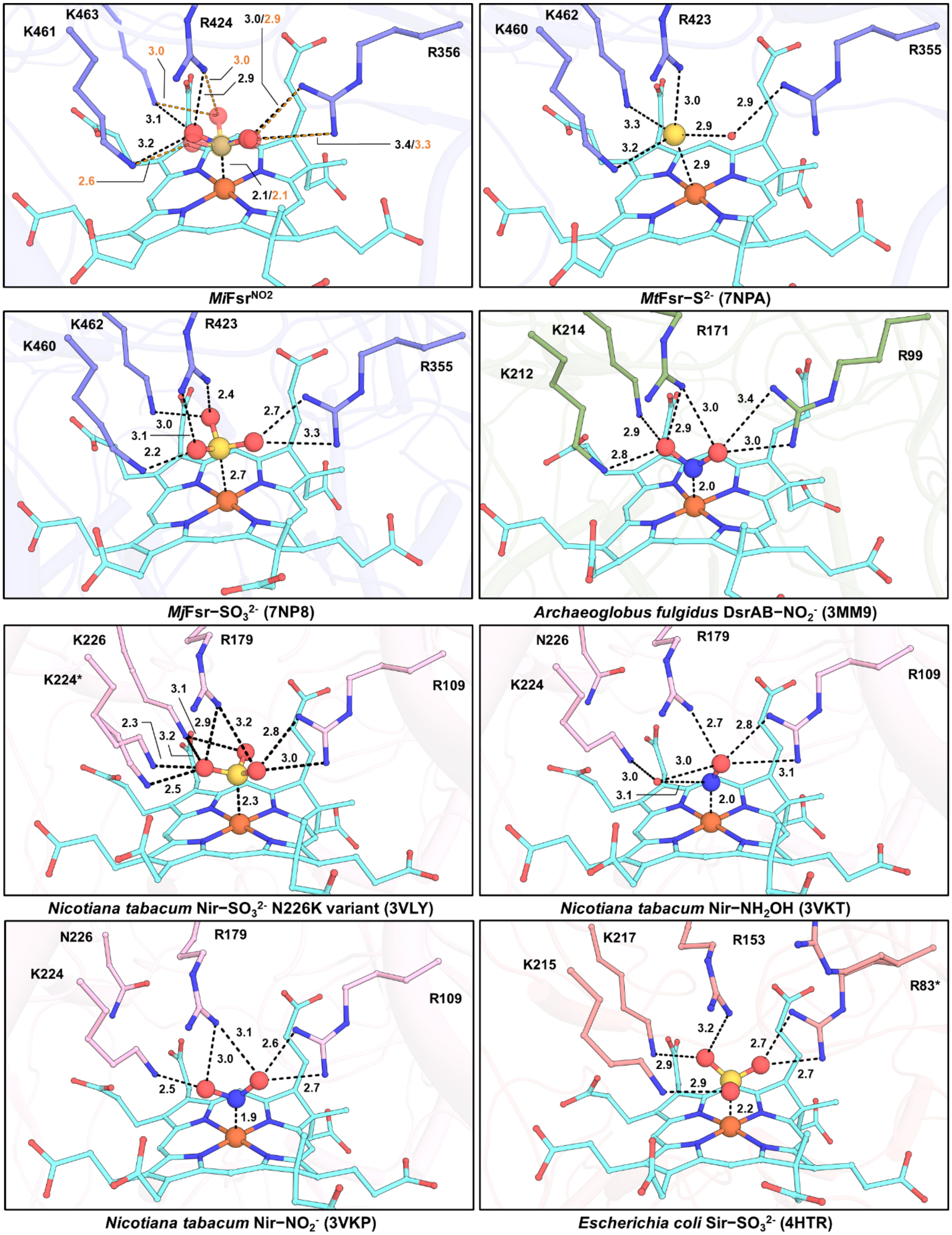

Fig. S6. Close-up of the NO_2^-/SO_3^{2-} reductase active site with ligands as spheres and the interacting residues in sticks. Distances in Å are labelled. For *Af*DsrAB, the numbering corresponds to the protein sequence and not the sequence from the structure. Residues marked with a "*" are shown with a double conformation. Interacting waters were removed for clarification, except for the bridging water in *Mt*Fsr and *Nt*Nir-NH_2OH.

References

1. Maslać N, Törer MR, Bolte P, Wagner T. Molecular basis of N_2 fixation in a hyperthermophilic archaeon. bioRxiv. 2025:2025.10.10.681579.
2. Dias JM, Than ME, Humm A, Huber R, Bourenkov GP, Bartunik HD, et al. Crystal structure of the first dissimilatory nitrate reductase at 1.9 Å solved by MAD methods. Structure. 1999;7(1):65-79.
3. Robert X, Guillon C, Gouet P. FoldScript: a web server for the efficient analysis of AI-generated 3D protein models. Nucleic Acids Res. 2025;53(W1):W277-W82.
4. Jespersen M, Pierik AJ, Wagner T. Structures of the sulfite detoxifying F_{420}-dependent enzyme from *Methanococcales*. Nat Chem Biol. 2023;19(6):695-702.
